# N^6^-methyladenosine-modified circIGF2BP3 inhibits CD8^+^ T-cell responses to facilitate tumor immune evasion by promoting the deubiquitination of PD-L1 in non-small cell lung cancer

**DOI:** 10.1186/s12943-021-01398-4

**Published:** 2021-08-20

**Authors:** Zhenchuan Liu, Tingting Wang, Yunlang She, Kaiqing Wu, Shaorui Gu, Lei Li, Chenglai Dong, Chang Chen, Yongxin Zhou

**Affiliations:** 1 Department of Thoracic Surgery, Shanghai Tongji Hospital, School of Medicine, Tongji University, Xincun Rd. 389, Shanghai, 200065 People’s Republic of China; 2 Department of Thoracic Surgery, Shanghai Pulmonary Hospital, School of Medicine, Tongji University, Zhengmin Rd. 507, Shanghai, 200443 People’s Republic of China

**Keywords:** Circular RNA, Non-small-cell lung carcinoma, Plakophilin 3, PD-L1, Immune escape

## Abstract

**Background:**

An in-depth understanding of immune evasion mechanisms in tumors is crucial to overcome resistance and enable innovative advances in immunotherapy. Circular RNAs (circRNAs) have been implicated in cancer progression. However, much remains unknown regarding whether circRNAs impact immune escape in non-small-cell lung carcinoma (NSCLC).

**Methods:**

We performed bioinformatics analysis to profile and identify the circRNAs mediating immune evasion in NSCLC. A luciferase reporter assay, RNA immunoprecipitation (RIP), RNA pulldown assays and fluorescence in situ hybridization were performed to identify the interactions among circIGF2BP3, miR-328-3p, miR-3173-5p and plakophilin 3 (PKP3). In vitro T cell-mediated killing assays and in vivo syngeneic mouse models were used to investigate the functional roles of circIGF2BP3 and its downstream target PKP3 in antitumor immunity in NSCLC. The molecular mechanism of PKP3-induced PD-L1 upregulation was explored by immunoprecipitation, RIP, and ubiquitination assays.

**Results:**

We demonstrated that circIGF2BP3 (hsa_circ_0079587) expression was increased in NSCLC and negatively correlated with CD8^+^ T cell infiltration. Functionally, elevated circIGF2BP3 inactivated cocultured T cells in vitro and compromised antitumor immunity in an immunocompetent mouse model, and this effect was dependent on CD8^+^ T cells. Mechanistically, METTL3 mediates the N^6^-methyladenosine (m^6^A) modification of circIGF2BP3 and promotes its circularization in a manner dependent on the m^6^A reader protein YTHDC1. circIGF2BP3 competitively upregulates PKP3 expression by sponging miR-328-3p and miR-3173-5p to compromise the cancer immune response. Furthermore, PKP3 engages with the RNA-binding protein FXR1 to stabilize OTUB1 mRNA, and OTUB1 elevates PD-L1 abundance by facilitating its deubiquitination. Tumor PD-L1 deletion completely blocked the impact of the circIGF2BP3/PKP3 axis on the CD8^+^ T cell response. The inhibition of circIGF2BP3/PKP3 enhanced the treatment efficacy of anti-PD-1 therapy in a Lewis lung carcinoma mouse model. Collectively, the PKP3/PD-L1 signature and the infiltrating CD8^+^ T cell status stratified NSCLC patients into different risk groups.

**Conclusion:**

Our results reveal the function of circIGF2BP3 in causing immune escape from CD8^+^ T cell-mediated killing through a decrease in PD-L1 ubiquitination and subsequent proteasomal degradation by stabilizing OTUB1 mRNA in a PKP3-dependent manner. This work sheds light on a novel mechanism of PD-L1 regulation in NSCLC and provides a rationale to enhance the efficacy of anti-PD-1 treatment in NSCLC.

**Supplementary Information:**

The online version contains supplementary material available at 10.1186/s12943-021-01398-4.

## Background

Lung cancer remains the most common malignancy and is the leading cause of cancer-related death globally [1]. Non-small-cell lung cancer (NSCLC), which primarily comprises lung adenocarcinoma (LUAD) and lung squamous cell carcinoma (LUSC), is the major type of primary lung cancer [2]. Most patients with advanced NSCLC have a poor prognosis due to the compromised efficacy of traditional therapy, metastasis at diagnosis and high relapse after treatment [3]. Recently, immune checkpoint blockade therapies (ICBs) blocking programmed cell death protein 1 (PD-1) and its ligand (PD-L1) have shown tremendous benefit for the treatment of advanced NSCLC [4]. However, many patients respond poorly to ICBs and develop resistance to PD-1 therapy [5, 6]. Understanding the molecular mechanism of PD-L1 regulation in NSCLC is helpful for improving the clinical effect of PD-L1/PD-1 therapy [7, 8].

Circular RNAs (circRNAs) are abundant and highly conserved noncoding RNAs (ncRNAs) and are characterized by covalent closed-loop structures [9, 10]. circRNAs are generated by back-splicing from their host genes and are highly stable due to the absence of 5′ caps and 3′ tails [11]. circRNAs can interact with miRNAs and thereby regulate miRNA-targeted gene expression by competitively binding to miRNA response elements [12]. Accumulating evidence has confirmed the roles of circRNAs in regulating the proliferation, metastasis, stemness and resistance to therapy of NSCLC [13–15]. For instance, circPPKCI accelerates the proliferation and progression of NSCLC by sponging miR-545 and miR-589, thus promoting transcription factor E2F7 expression [16], while hsa_circ_0014235 increases the resistance of NSCLC cells to cisplatin by enhancing CDK4 expression [17]. However, the biological implications of circRNAs in regulating antitumor immunity in NSCLC remain unclear.

Increasing evidence suggests that m^6^A modification, one of the major posttranscriptional modifications of eukaryotic RNAs, participates in various aspects of RNA homeostasis [18]. Importantly, dysregulated m^6^A profiles have been implicated in the carcinogenesis and progression of NSCLC. METTL3 (methyltransferase like 3), a component of the methyltransferase complex that catalyzes N^6^ methylation, is elevated in NSCLC and induces gefitinib resistance in NSCLC cells by enhancing autophagy-related gene expression [19]. METTL3 also facilitates NSCLC metastasis by promoting the translation of m^6^A-modified YAP [20]. However, the function of m^6^A modification and the role of m^6^A-modified circRNAs in regulating the antitumor immunity of NSCLC remain elusive.

In this work, we found that circIGF2BP3, a circRNA derived from a back-splicing event between exons 4 and 13 of IGF2BP3, is markedly overexpressed and compromises antitumor immunity in NSCLC. We determined that high circIGF2BP3 expression is due to enhanced circularization resulting from increased m^6^A levels of the circIGF2BP3 transcript in a METTL3-dependent manner. Moreover, circIGF2BP3 competitively upregulates plakophilin 3 (PKP3) expression by sponging miR-328-3p and miR-3173-5p, and the immunosuppressive effect of circIGF2BP3 is dependent upon its downstream target PKP3. PKP3 is a member of the arm repeat family of catenin proteins and serves as a structural component of desmosomes, mediating cell-cell adhesion and communication [21]. Recent studies have shown that PKP3 participates in the progression and metastasis of ovarian cancer, prostate cancer and nasopharyngeal carcinoma [22–24], but its molecular mechanism underlying the regulation of antitumor immunity remains unclear. We showed that PKP3 forms protein-RNA complexes with FXR1, which increases the mRNA stability of the deubiquitinase OTUB1. PKP3-mediated OTUB1 upregulation in turn alleviates PD-L1 ubiquitination and subsequent proteasomal degradation. We further explored the regulatory effect of the circIGF2BP3/PKP3 axis on PD-L1 expression and its impact on tumor growth and antitumor immunity in vitro and in vivo. Our work provides a novel mechanism of immune escape in NSCLC and identifies a promising new pharmaceutical intervention target for NSCLC patients receiving anti-PD-1 treatment.

## Materials and methods

### Human samples

A total of 68 NSCLC patients (termed cohort I) who were diagnosed at Shanghai Tongji Hospital in 2014–2015 were enrolled in this study. NSCLC tissues and paired adjacent nontumorous tissues frozen in liquid nitrogen and corresponding archived paraffin-embedded specimens were collected. None of the patients in this study received any systemic treatment before the samples were collected. The patients enrolled in this study were continuously followed over time.

This study was approved by the Medical Ethics Committee of Shanghai Tongji Hospital. Written informed consent was obtained from each patient prior to this study.

### Cell culture

HEK293T cells, human LUAD cells (A549, NCI-H1650 and NCI-H1975), human LUSC cells (SW900, SK-MES-1, NCI-H1703 and NCI-H520), a normal lung epithelial cell line (BEAS-2B) and a bronchial epithelial cell line (16HBE) were obtained from the Cell Bank of the Chinese Academy of Sciences (Shanghai, China). SW900 cells were cultured in Leibovitz’s L-15 medium supplemented with 10% fetal bovine serum (FBS). A549 cells were cultured in F-12 K medium supplemented with 10% FBS. HEK293T and SK-MES-1 cells were cultured in minimal essential media supplemented with 10% FBS. BEAS-2B cells were cultured using a BEGMTM BulletKitTM (Lonza, GA, USA). 16HBE, NCI-H1650, NCI-H1975, NCI-H1703 and NCI-H520 cells were cultured in RPMI-1640 medium supplemented with 10% FBS. All cells were authenticated by the short tandem repeat method and were checked for mycoplasma contamination.

### Plasmids

To generate a lentivirus-based expression system, short hairpin RNAs (shRNAs) targeting PKP3 and circIGF2BP3 were synthesized and inserted into the shRNA expression vector pLVX-shRNA (TaKaRa Bio, Beijing, China). Full-length cDNA of PKP3 was amplified by RT-PCR using total RNA from HEK293T cells and then subcloned into the pLVX-Puro vector (TaKaRa Bio).

For the luciferase reporter assay, versions of circIGF2BP3 and PKP3 with mutated miRNA binding sites were generated with a mutagenesis kit (Vazyme, Nanjing, China). Wild-type and mutant 3′ untranslated regions (UTRs) of PKP3 were synthesized and subcloned into the pmirGLO3 vector (Promega, WI, USA). The wild-type and mutant linear forms of circIGF2BP3 were synthesized and subcloned into the psiCHECK2 vector (Promega). The sequences of specific mutations in miRNA binding sites are illustrated in Fig. S5.

For circRNA overexpression, the linear sequence of circIGF2BP3 flanked by the cyclization sequence was amplified and inserted into the pLCDH-ciR plasmid. circIGF2BP3-si-mut was constructed based on the same method except using different primers. circIGF2BP3-m^6^A-mut, a plasmid with a mutated sequence in its m^6^A site, was generated by site-directed mutagenesis (Vazyme). The sequences of specific mutations in short interfering RNA (siRNA)-targeted sites and m^6^A sites are illustrated in Fig. S2 and Fig. S3, respectively.

The cDNA of PKP3ΔC (aa 1–515) and full-length cDNAs of PKP3, OTUB1, PD-L1 and ubiquitin were amplified by RT-PCR using total RNA from HEK293T cells. Next, myc-ubiquitin and myc-PKP3ΔC were subcloned into a pRK5 vector, Flag-PD-L1 was subcloned into the pRK5 vector, and V5-PKP3 and V5-OTUB1 were subcloned into the pRK5 vector.

siRNAs targeting IGF2BP3, METTL3, METTL14, WTAP, YTHDC1, OTUB1, CSN5, STUB1, USP22, SPOP, FBXO38, HRD1 and FXR1 were synthesized by Sangon Biotech (Shanghai, China). The miR-328-3p mimics, miR-3173-5p mimics, miR-195-5p mimics, miR-499a-5p mimics, miR-497-5p mimics, miR-16-5p mimics, miR-15b-5p mimics, miR-424–5p mimics, miR-15a-5p mimics and negative control mimics were synthesized by GenePharma (Shanghai, China).

sgRNAs targeting mouse PD-L1 were designed using CRISPR design (http://crispr.mit.edu) and synthesized by Sangon Biotech. The gRNA was then ligated and cloned into lentiCRISPR (Cat# 52961, Addgene). The lentiCRISPR-gRNA construct and its packaging plasmids were cotransfected into HEK293T using Lipofectamine 3000 (Invitrogen, MA, USA). The cells were cultured in DMEM. The collected viral particles in the medium were concentrated and transduced into Lewis lung cancer (LLC) cell lines using polybrene.

All of the oligonucleosides used in this study are listed in Table S3.

### RNA extraction, RT-PCR and qRT-PCR

For RNA extraction, total RNA was isolated using TRIzol (Invitrogen) and reverse transcribed using the PrimeScript RT Reagent Kit (TaKaRa Bio). For the subcellular fractionation of RNA, NE-PER nuclear and cytoplasmic extraction reagents (Pierce, IL, USA) were used to separate the nuclear and cytoplasmic fractions of the indicated cells. qRT-PCR using SYBR Premix Ex Taq (TaKaRa Bio) was employed to identify the levels of the RNAs of interest. The results were normalized to those of GAPDH. The mRNA levels were calculated using the 2^–ΔΔCt^ method. The primers used in this study are listed in Table S3.

### RNase R treatment

Total RNA of the indicated cells was treated with or without RNase R (5 U/μg RNA) for 30 min at 37 °C. After purification, the expression of circIGF2BP3 was determined by quantitative real-time PCR (qRT-PCR).

### Actinomycin D assay

Cells were exposed to actinomycin D (10 μg/ml) for the indicated times (0, 8, 24, and 48 h); the total RNA of cells was then collected. The expression of circIGF2BP3 was determined by qRT-PCR.

### Luciferase reporter assay

For the luciferase assay, cells were transfected with the indicated constructs using Lipofectamine 3000 (Thermo Scientific, MA, USA). The luciferase activity was measured with a Dual-Glo Luciferase Assay System (Promega) and an illuminometer. Renilla luciferase intensity was used as a control to normalize the firefly luciferase intensity.

### Immunofluorescence assay and fluorescence in situ hybridization (FISH)

For cell-based immunofluorescence (IF), NSCLC cells or adhered cocultured peripheral blood monocyte cells (PBMCs) were fixed in 4% paraformaldehyde, permeabilized with 0.1% Triton X-100, and blocked with 3% bovine serum albumin (BSA). For PD-L1 and Ki-67 staining, the indicated cells were incubated with primary antibodies against PD-L1 and Ki-67 (Abcam, Cambridge, UK) at 4 °C overnight. The cells were then incubated with Alexa Fluor-conjugated secondary antibodies (Abcam). For TUNEL staining, the cells were incubated with TUNEL reaction reagent (Beyotime, Beijing, China). For the PD-1 binding assay, NSCLC cells were incubated with recombinant human PD-1 Fc protein (Abcam), followed by incubation with anti-human IgG/Alexa Fluor 647 dye (Abcam). Nuclei were counterstained with DAPI (Sigma-Aldrich, Munich, Germany).

For slide-based IF, paraffin-embedded sections of patient or mouse samples were baked, deparaffinized, and rehydrated, followed by antigen retrieval by treating with 1× EDTA at 98 °C for 10 min. The samples were then washed, blocked with 5% BSA, and permeabilized with 0.1% Triton X-100 for 1 h at room temperature. The sections were incubated with primary antibodies against group 1 (PD-L1, CD8α and PKP3) or group 2 (PKP3 and OTUB1) at 4 °C overnight. Following rinsing with PBS, the samples were incubated with secondary antibody (anti-mouse Alexa Fluor 647/Alexa Fluor 488 or anti-rabbit Alexa Fluor 647/Alexa Fluor 488, Abcam) for 2 h at room temperature. In each round, only one primary antibody was added. Antigen retrieval was performed during each round. Nuclei were counterstained with DAPI. After staining, the slides were coverslipped with anti-fade medium and stored in the dark for further imaging.

For FISH, digoxin-labeled probes specific to circIGF2BP3, miR-328-3p and miR-3173-5p were synthesized by Sangon Biotech. NSCLC cells were fixed and prehybridized in prehybridization solution (PBS with 0.5% Triton X-100). Next, the cells were incubated with the digoxin-conjugated probes in hybridization solution (salmon sperm DNA, yeast tRNA, 40% formamide, 10% dextran sulfate, 1× Denhardt’s solution and 4× SSC) at 58 °C overnight and subsequently with anti-DIG-FITC/anti-DIG-Cy5 for 2 h at room temperature. Nuclei were counterstained with DAPI.

Images were acquired and analyzed by fluorescence microscopy (Olympus, Tokyo, Japan) and ImageJ, respectively.

### Flow cytometry

To assess apoptosis, NSCLC cells were double-stained with annexin V-FITC and propidium iodide (PI) (BD Biosciences, CA, USA) according to the manufacturer’s instructions, and staining was detected using a CytoFLEX flow cytometer (Beckman Coulter, CA, USA).

For mouse samples, the tumor tissues were first processed into single-cell suspensions by grinding and filtration. The samples were then blocked with CD16/CD32 antibody (BioLegend, CA, USA), and dead cells were excluded using the Zombie Red Fixable Viability Kit (BioLegend). Tregs (CD4^+^/Foxp3^+^), myeloid-derived suppressor cells (MDSCs, CD11b^+^) and M2-like tumor-associated macrophages (M2-TAMs, CD68^+^CD206^+^) infiltrating the tumor regions were labeled by staining the cells with the indicated antibody. To determine activated CD8^+^ T cells in the tumor samples, cells were stained with CD45, CD3 and CD8 (Abcam). After permeabilization with a Cytofix/Cytoperm Kit (BD Biosciences), the cells were incubated with the following antibodies: anti-IFN-γ and anti-TNF-α from Miltenyi Biotec (Germany) and anti-perforin and anti-granzyme B from eBioscience (CA, USA).

To determine the expression of circIGF2BP3/PKP3 in immune cells, CD45^+^, CD3^+^, CD4^+^ and CD8^+^ cells in PBMCs were sorted by flow cytometry and subjected to qPCR. The cells were labeled with anti-PD-L1 antibody to determine PD-L1 expression on the cell surface. Flow cytometric analysis was conducted using a CytoFLEX flow cytometer. All data were analyzed and plotted using FlowJo (TreeStar, OR, USA). The antibodies used in this study are listed in Table S1.

### Cell transfection

Cells at 80% confluence were transfected with the oligonucleotides (siRNA and miRNA mimics) and plasmid-based constructs using Lipofectamine 3000 (Invitrogen). After 24 h incubation, the cells were collected for subsequent experiments.

For the transfection of lentivirus-based constructs, cells at 80% confluence were incubated for 24 h in medium containing concentrated viral particles and polybrene (Sigma-Aldrich). The transfected cells were allowed to grow for another 2 days and then selected with puromycin (1 μg/ml) (Sigma-Aldrich) for 1 week. The transfection efficiency was validated by qRT-PCR or western blotting.

### T cell-mediated tumor cell killing assay

Human PBMCs were cultured in RPMI-1640 medium and activated with Dynabeads™ Human T-Activator CD3/CD28 (Gibco, CA, USA) for 1 week according to the manufacturer’s instructions. NSCLC cells were seeded into 12-well plates at a cell-dependent concentration. After 24 h, activated PBMCs were cocultured with adhered NSCLC cells for 48 h at a ratio of 3:1. After 48 h of incubation, cell debris was removed, PBMCs were collected, and NSCLC cells were harvested and labeled with annexin V and PI for fluorescence-activated cell sorting (FACS) analysis.

### Protein extraction, western blotting, immunoprecipitation (IP) and RNA immunoprecipitation (RIP)

Total protein was extracted from tissues and cells using RIPA lysis buffer (Solarbio, Beijing, China) supplemented with protease inhibitor and phosphatase inhibitor. The concentration of the extracted proteins was determined using the bicinchoninic acid method (Solarbio).

For western blotting, the extracted proteins were separated on 10% sodium dodecyl sulfate (SDS)-PAGE gels and transferred to polyvinylidene fluoride membranes (Invitrogen). The membranes were blocked in blocking buffer (EpiZyme, Shanghai, China) and incubated with the indicated primary antibodies overnight at 4 °C. The membranes were then incubated with horseradish peroxidase (HRP)-conjugated secondary antibodies (Abcam) for 1 h at room temperature and washed with TBST. The blots were developed via the enhanced chemiluminescence method (EpiZyme).

For the ubiquitination assay, the indicated cells were transfected with various constructs together with Myc-ubiquitin and Flag-PD-L1, treated with MG132, and lysed using RIPA lysis buffer. Ubiquitination was assessed by IP with an antibody against the Flag tag, followed by western blotting with an anti-myc antibody.

For IP, the extracts of the indicated cells were precleared using protein A-agarose, and IP was performed by incubating the supernatants with primary antibodies against PKP3 and myc tag for 1 h at 4 °C. The samples were then incubated with protein A/G overnight at 4 °C. After centrifugation, the protein A-agarose-antigen-antibody complexes were washed with lysis buffer and resuspended in loading buffer (Solarbio). The proteins were separated by SDS-PAGE and detected by western blotting. IP with a rabbit IgG isotype was conducted as a negative control.

The RIP assay was conducted using the Magna RIP RNA-Binding Protein IP Kit (Millipore, MA, USA) according to the manufacturer’s protocol. Protein A/G agarose beads coated with primary antibodies against IgG, PKP3, myc tag and AGO2 (Abcam) were incubated with the cell lysates in RIP buffer at 4 °C overnight. The protein-RNA complexes were washed, digested with proteinase K, and recovered using TRIzol. The abundance of protein-bound RNA was determined by qRT-PCR and normalized to the input. The antibodies used in this study are listed in Table S1.

### Methylated RNA immunoprecipitation (MeRIP)

Total RNA was extracted using TRIzol. Fifty nanograms of total RNA was removed as an input control, and primary antibodies against IgG and m^6^A (Abcam) were added to the remaining RNA in buffer containing 150 mM NaCl, 0.1% NP-40, 10 mM Tris and RNase inhibitor. The primary antibody pulldown portion was collected via IP with Dynabeads® Protein A (Invitrogen), washed with elution buffer, and recovered by ethanol precipitation. The relative interaction between RNA and protein was determined by qRT-PCR and normalized to the input.

### CCK-8 assay

Cell viability was assessed using the Cell Counting Kit-8 assay (Dojindo, Tokyo, Japan) according to the manufacturer’s instructions. Briefly, the indicated cells were seeded into 96-well plates at a cell-dependent concentration. After culturing for different time periods, the cells were incubated with CCK-8 reagent for 2 h. The absorbance of each well was measured at 450 nm.

### Immunohistochemistry (IHC)

Paraffin-embedded slides were deparaffinized, rehydrated and then subjected to antigen retrieval by treating with 1× EDTA at 98 °C for 10 min. The slides were further incubated in 3% H_2_O_2_ solution to block endogenous peroxidase. After blocking with 10% goat serum, the tissues were incubated with primary antibodies against PD-L1, PKP3, and CD8 at 4 °C overnight. After rinsing with PBS, the slides were incubated with biotin-conjugated secondary antibody, washed, and incubated with HRP-conjugated streptavidin. Immunoreactive proteins were detected by 3,3′-diaminobenzidine staining (Zhongshan Goldenbridge Biotechnology Company, Beijing, China). Hematoxylin was used to counterstain the slides. The abundance of the target protein was scored semiquantitatively based on the product of the staining intensity score (0, negative staining; 1, weak staining; and 2, strong staining) and the proportion of positively stained cells score (0, no positive cells, 1, < 50% positive cells; and 2, > 50% positive cells). A final score ≥ 3 was considered to indicate high expression, and a final score < 3 was considered to indicate low expression. Two pathologists independently scored each sample to avoid bias.

### Protein stability

Cells were exposed to cycloheximide (CHX, 100 μg/ml) for the indicated times (0, 3, and 12 h). The cells were then harvested, and total proteins were obtained. The abundance of PD-L1 was measured by western blotting.

### Biotin-coupled miRNA pulldown

For this assay, 3′ biotinylated miR-328-3p, miR-3173-5p and control mimics synthesized by Sangon Biotech were transfected into NSCLC cells using Lipofectamine 3000 (Invitrogen). Forty-eight hours after transfection, the cells were lysed in lysis buffer (protease inhibitor, 50 U/ml RNaseOUT, and 0.5% NP-40 in TE (pH 7.5)). The biotin-coupled RNA complex was washed and incubated with streptavidin-coated magnetic beads (Invitrogen), followed by magnetic separation and centrifugation. The abundance of circIGF2BP3 and PKP3 in the biotin-coupled RNA complex was determined by qRT-PCR.

### In vivo study

Four-to-five-week-old female C57BL/6 mice were purchased from SLAC (Shanghai, China) and housed under pathogen-free conditions. PD-L1-deficient C57BL/6 (PD-L1 knockout (KO)) mice were purchased from the Shanghai Model Organism Center (Shanghai, China) and maintained as a homozygous line. A total of 10^6^ LLC cells transfected with the following constructs were injected subcutaneously into C57BL/6 mice. The treatments are listed as follows:

(1) circIGF2BP3-OE LLC cells or control (CTRL) cells were inoculated into mice with CD8α neutralizing monoclonal antibody (mAb) or control IgG isotype administration to evaluate the immunosuppressive effect of circIGF2BP3 and whether this effect was dependent on CD8^+^ tumor-infiltrating lymphocytes (TILs). (2) circIGF2BP3-OE LLC cells and CTRL cells with or without PKP3 silencing were utilized to explore the role of the circIGF2BP3/PKP3 axis in immune escape. (3) sgPD-L1 LLC cells and CTRL cells with or without PKP3 overexpression were generated to test whether the immune-suppressing roles of PKP3-mediated effects were dependent on PD-L1. (4) A mouse anti-PD-1 mAb or control IgG isotype was administered to mice implanted with PKP3-silenced LLC cells to observe whether PKP3 knockdown enhanced the efficacy of anti-PD-1 treatment. (5) PKP3-silenced LLC cells were implanted into wild-type (WT) or PD-L1 KO C57BL/6 mice followed by treatment with or without anti-PD-1 mAb to explore the impact of PD-L1 expressing in stromal cells residing in the tumor microenvironment (TME) on CD8^+^ T cell-mediated antitumor immunity. (6) A mouse anti-PD-1 mAb or control IgG isotype was administered to C57BL/6 mice implanted with CTRL, circIGF2BP3-OE or PKP3-OE LLC cells to test the impact of circIGF2BP3 overexpression or PKP3 overexpression on the efficacy of anti-PD-1 treatment.

Mice were pooled and randomized when the tumors were palpable. CD8α mAb, anti-PD-1 mAb or the corresponding IgG2 isotype was administered intraperitoneally every 3 days at a concentration of 100 μg/mouse. Tumor volume was measured every 3 days using a caliper and was calculated as follows: length × width^2^ × 0.5. 15 or 18 days after the mice were separated into groups, the mice were sacrificed, and tumor samples were harvested and prepared for analysis. This in vivo study was approved by the Animal Care and Use Committee of Shanghai Tongji Hospital and was conducted in accordance with ethical standards.

### Bioinformatics

Gene Expression Omnibus (GEO) data sets (https://www.ncbi.nlm.nih.gov/geo/) (GSE126533) were used to analyze the expression of circRNAs and mRNAs in NSCLC samples. Weighted gene coexpression network analysis (WGCNA) was performed to enrich modules that were associated with CD8^+^ T cell infiltration [25]. CIBERSORT (https://cibersortx.stanford.edu) and TIMER 2.0 (http://timer.cistrome.org/) were used to estimate the proportion of infiltrated CD8^+^ T cells in NSCLC tissues from GSE126533 and select candidate mRNAs that negatively regulated CD8^+^ T cell infiltration in NSCLC tissues from The Cancer Genome Atlas (TCGA) data set. Visualization of the competing endogenous RNA (ceRNA) network and coexpression network comprising circIGF2BP3 and associated mRNAs was carried out using Cytoscape software (https://cytoscape.org). The mRNA expression data of NSCLC patients in the TCGA database (https://portal.gdc.cancer.gov/) were used to analyze the expression profile of mRNAs and the correlation between PKP3 and OTUB1. StarBase (http://starbase.sysu.edu.cn/) was used to construct a ceRNA network and predict candidate miRNAs sponged by circIGF2BP3 and candidate miRNAs targeting the 3′ UTR of PKP3. TIMER 2.0 was employed to explore the relationship between METTL3 expression and CD8^+^ T cell infiltration. Kaplan-Meier Plotter (http://kmplot.com) was used to explore the prognostic value of PKP3, miR-328-3p, miR-3173-5p and OTUB1 in NSCLC. RMBase 2.0 (http://rna.sysu.edu.cn/rmbase) and SRAMP (http://www.cuilab.cn/sramp/) were used to screen candidate m^6^A sites in circIGF2BP3. The expression data for differential analysis and correlation analysis were log_2_(TPM + 1) transformed. Raw data were collected from these data sets and manipulated, analyzed, and presented via R 3.6.0 and GraphPad Prism 7.0. All the code supporting the conclusions of the study is available from the corresponding author upon reasonable request.

### Statistics

The data are presented as the mean ± SD. Data analysis was conducted using GraphPad Prism 7.0. Unpaired or paired 2-tailed Student’s t tests were conducted for comparisons between 2 groups. One-way ANOVA with Tukey’s post hoc test was performed to compare more than 2 groups. Correlations between the expression levels of different factors were assessed by Pearson correlation coefficient and Spearman correlation coefficient analyses. The survival data were analyzed using the log-rank test and are presented as Kaplan-Meier survival curves. A Cox proportional hazards model was employed to perform univariate and multivariate analyses. All data were tested beforehand to meet the assumptions of the statistical analysis (e.g., normal distribution, adequate statistical power, and homogeneity of variance). All experiments were repeated at least three times. Differences with *P* < 0.05 were considered significant.

## Results

### Identification and characterization of a novel circRNA, circIGF2BP3, which suppresses CD8^+^ TIL infiltration in NSCLC

**Fig. 1:**
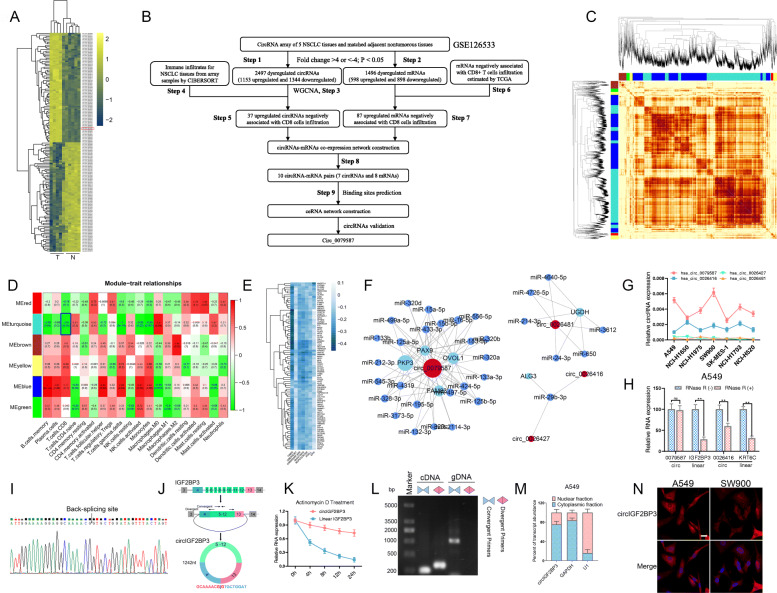
Identification of circIGF2BP3 as a candidate circRNA that inhibits CD8^+^ TIL infiltration and antitumor immunity in NSCLC. **A** Heatmap showing the top 75 most upregulated circRNAs and the 75 most downregulated circRNAs in NSCLC tissues (T) and paired nontumor tissues (N) analyzed in GSE126533. hsa_circ_0079587 (circIGF2BP3) is marked with a red box. **B** Flowchart of the steps utilized in the present study to identify and validate circRNAs in NSCLC. **C** Topological overlap matrix (TOM) and clustering diagram of circRNAs presenting similarity based on topological overlap combined with assigned module color. **D** The relationship between circRNA modules and clinical traits. Each cell represents the correlation coefficient (upper) and *p*-value (lower) between the corresponding module eigengene and clinical trait. The eigengene of the turquoise module marked in the blue box is negatively correlated with CD8^+^ TIL infiltration. **E** Heatmap showing 87 mRNAs that were both upregulated in NSCLC and negatively correlated with CD8^+^ TIL infiltration predicted by at least 4 algorithms. **F** The constructed ceRNA network of circRNA-miRNA-mRNA interactions. Nodes in red, dark blue and light blue represent circRNAs, miRNAs and mRNAs, respectively. **G** The relative expression of the four indicated circRNAs in different NSCLC cell lines detected by qRT-PCR. GAPDH was used as an internal control. **H** RNase R treatment and qRT-PCR analyses were performed to validate the corresponding circRNAs. **I** Sanger sequencing was conducted to identify the back-splicing site of circIGF2BP3. **J.** Diagram illustrating that circIGF2BP3 is generated from back-splicing between exons 4 and 13 of linear IGF2BP3. **K** Time-course qRT-PCR analyses of the relative abundance of circIGF2BP3 and linear IGF2BP3 in A549 cells treated with actinomycin D (10 μg/ml). **L** qPCR analysis of circIGF2BP3 and its linear form in cDNA and gDNA amplified by convergent and divergent primers, respectively. **M** qRT-PCR analysis of circIGF2BP3 abundance in the cytoplasmic and nuclear fractions of A549 cells. GAPDH and U1 were used as positive controls in the cytoplasm and nucleus, respectively. **N** FISH indicating that circIGF2BP3 was mainly located in the cytoplasm. Scale bars, 20 μm. Data represent the mean ± SD. *P < 0.05, **P < 0.01, ***P < 0.001. *P* values were determined by unpaired Student’s t test (**H** and **K**)

To characterize dysregulated circRNAs and mRNAs in NSCLC, we initially analyzed microarray data containing the expression profiles of circRNAs and mRNAs in 5 paired NSCLC tissues (GSE126533) [26] (Fig. 1A). By applying the criteria (fold change > 4, p.adjust < 0.05), 2497 dysregulated circRNAs and 1496 dysregulated mRNAs were identified (Step 1 and Step 2, Fig. 1B, Table S4–5). To further screen candidate circRNAs mediating immune escape, these dysregulated circRNAs were subjected to WGCNA, and six circRNA modules were identified (Step 3, Fig. 1C, Fig. S1A). We estimated the relative abundance of CD8^+^ TILs in samples from GSE126533 by applying the CIBERSORT algorithm, as cytotoxic CD8^+^ TILs are the major contributor to the successful elimination of cancer cells [27] (Step 4, Fig. S1B). Trait-module relationship analysis revealed that the expression of circRNAs in the turquoise module was negatively correlated with CD8^+^ TIL infiltration, and 37 hub circRNAs were identified from the turquoise module based on intramodular analysis (gene significance > − 0.96, module membership value > 0.96 and q weighted < 0.01) (Step 5, Fig. 1D, Fig. S1C-D, Table S6). It has been acknowledged that circRNAs primarily act as miRNA sponges to positively regulate miRNA-targeted gene expression [28, 29]. To identify potential mRNAs regulated by circRNAs, we employed 6 distinct algorithms (TIMER, CIBERSORT, XCELL, EPIC, QUANTISEQ and MCPCOUNTER) integrated and implemented in TIMER 2.0 (http://timer.cistrome.org) to estimate the immune infiltrates in NSCLC samples of the TCGA data set. This, which identified 327 genes negatively correlated with CD8^+^ TIL infiltration based on prediction by at least 4 algorithms (Step 6). Among these, a total of 87 genes were upregulated in GSE126533 (Step 7, Fig. 1E). We next built a coexpression network between the 37 upregulated CD8^+^ TIL-related circRNAs and the 87 upregulated genes that possibly inhibited CD8^+^ TIL infiltration and identified 10 circRNA-mRNA interactions (Step 8). Finally, we constructed a circRNA-miRNA-mRNA regulatory network, and circRNA-miRNA pairs and miRNA-mRNA pairs were predicted by starBase (Step 9, Fig. 1F, Table S7). The network contained 4 circRNAs, 32 miRNAs and 6 mRNAs.

We next verified these circRNAs by qRT-PCR and RNase R treatment. Among the four circRNAs, only hsa_circ_0079587 (termed circIGF2BP3) was highly expressed in all NSCLC cell lines tested and was resistant to RNase R treatment (Fig. 1G-H). According to the annotation in circBase, circIGF2BP3 is formed by back-splicing of exons 4 and 13 of the IGF2BP3 mRNA and has a length of 1242 bp (Fig. 1J). Accordingly, Sanger sequencing confirmed the back-splicing site of circIGF2BP3 (Fig. 1I). Compared with its linear form, circIGF2BP3 was more stable after treatment with actinomycin D (Fig. 1K). Additionally, circIGF2BP3 could be amplified by divergent primers from cDNA but not gDNA (Fig. 1L). Finally, FISH and qRT-PCR of nuclear and cytoplasmic fractions revealed that circIGF2BP3 was mainly located in the cytoplasm (Fig. 1M-N). These results identified a highly stable circRNA in NSCLC cells derived from exons 4 to 13 of the IGF2BP3 gene locus.

### Elevated circIGF2BP3 expression predicts poor prognosis in NSCLC patients

**Fig. 2:**
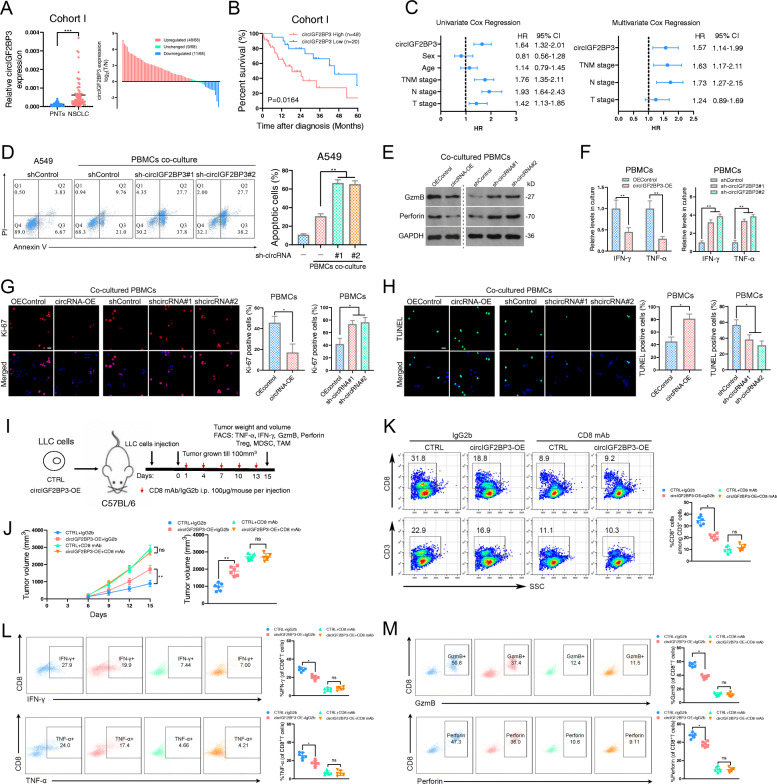
circIGF2BP3 is overexpressed in NSCLC and attenuates CD8^+^ T cell-mediated antitumor immunity. **A** The relative expression of circIGF2BP3 in 68 paired samples of NSCLC in cohort I from Tongji Hospital. GAPDH was used as an internal control. **B** Kaplan-Meier (K-M) plot showing the relationship between circIGF2BP3 levels and patient OS in cohort I from Tongji Hospital. **C** Cox regression analysis showed that circIGF2BP3 was an independent prognostic biomarker in NSCLC. **D** Representative images and statistical quantitation of FACS analysis of PBMC-mediated elimination of NSCLC cells, as determined by annexin V-FITC and propidium iodide (PI) double labeling. **E** Expression of perforin and GzmB in PBMCs after coculture with circIGF2BP3-knockdown A549 cells or circIGF2BP3-overexpressing SK-MES-1 cells. **F** The levels of IFN-γ and TNF-α secreted by PBMCs were detected by ELISA after coculturing with circIGF2BP3-knockdown A549 cells or circIGF2BP3-overexpressing SK-MES-1 cells. **G** Ki-67 staining of PBMCs after coculturing with circIGF2BP3-OE or circIGF2BP3-silenced NSCLC cells. Scale bars, 20 μm. **H** TUNEL staining of PBMCs after coculturing with circIGF2BP3-OE or circIGF2BP3-silenced NSCLC cells. Scale bars, 20 μm. **I** Schematic diagram exhibiting the grouping and treatment plan of the syngeneic mouse model: C57BL/6 mice were inoculated with 10^6^ CTRL or circIGF2BP3-OE LLC cells and received CD8 mAb treatment or IgG2b control treatment at the indicated time points. **J** Time-course evaluation of tumor volumes measured every 3 days. **K** Representative flow cytometry data for infiltrated CD8^+^ T cells in CTRL and circIGF2BP3-OE LLC xenografts. **L-M** Representative images and statistical quantitation of the FACS analysis of the percentage of IFN-γ^+^, TNF-α^+^, GzmB^+^ and perforin^+^ CD8^+^ TILs from CTRL and circIGF2BP3-OE LLC xenografts. Data represent the mean ± SD. *P < 0.05, **P < 0.01, ***P < 0.001. *P* values were determined by unpaired Student’s t test (**F, G** and **H**), paired Student’s t test (**A**), one-way ANOVA with Tukey’s post hoc test (**D, F, G, H, J, K, L** and **M**) and log-rank test (**B**)

We next explored the clinical significance of circIGF2BP3 in the progression of NSCLC. First, we found that circIGF2BP3 was preferentially expressed in NSCLC cells compared with immune cells (CD45^+^, CD3^+^, CD4^+^ and CD8^+^ cells) isolated from PBMCs of NSCLC patients. Furthermore, compared with normal lung or bronchial epithelial cells, the levels of circIGF2BP3 in NSCLC cell lines were significantly higher (Fig. S1E). Second, we confirmed the upregulation of circIGF2BP3 expression by qRT-PCR in 68 paired NSCLC tissues from our hospital (Fig. 2A). Third, high circIGF2BP3 expression was positively correlated with lymphatic metastasis and more advanced tumor stage in NSCLC patients (Table S8). Finally, survival analysis revealed that patients with high circIGF2BP3 expression had much shorter overall survival (OS, Fig. 2B). The univariate and multivariate regression analyses results demonstrated that high circIGF2BP3 expression was an independent prognostic marker for NSCLC (Fig. 2C). Collectively, these results suggest that circIGF2BP3 may play an oncogenic role in NSCLC.

### circIGF2BP3 suppresses the T cell response against NSCLC in vitro and in vivo

To confirm the role of circIGF2BP3 in antitumor immunity in NSCLC, we constructed two shRNAs (sh-circIGF2BP3#1 and sh-circIGF2BP3#2) targeting the back-splicing junction of circIGF2BP3 to silence circIGF2BP3 expression in NSCLC cells (Fig. S2A). To overexpress circIGF2BP3, exons 4 to 13 of IGF2BP3 were cloned into the pLCDH-ciR plasmid. On the basis of the results in Fig. 1G, A549 and SW900 cells were selected for the circIGF2BP3 loss-of-function assay, while H1650 and SK-MES-1 cells were selected for the gain-of-function assay. qRT-PCR confirmed elevated circIGF2BP3 expression in NSCLC cells transfected with the circIGF2BP3 overexpression plasmid and decreased expression in NSCLC cells following circIGF2BP3 knockdown; importantly, the linear IGF2BP3 mRNA levels did not change significantly (Fig. S2B-C). We performed a T cell-mediated killing assay to demonstrate that circIGF2BP3 silencing sensitized NSCLC cells to PBMC-mediated cytolysis, whereas circIGF2BP3 overexpression exhibited the opposite effect (Fig. 2D, Fig. S2D). We next explored whether circIGF2BP3 impacted the immunosuppressive effects of tumor cells on PBMCs. As shown in Fig. 2E-F, circIGF2BP3 depletion alleviated tumor cell-mediated immune suppression, as determined by the expression of perforin, granzyme B (GzmB) and secreted IFN-γ and TNF-α from cocultured PBMCs, whereas circIGF2BP3 overexpression compromised the expression or secretion of these immune effectors. Moreover, PBMCs cocultured with circIGF2BP3-silenced cells exhibited higher Ki-67 staining and a lower TUNEL ratio, while circIGF2BP3 overexpression dramatically inhibited PBMC proliferation and induced apoptosis (Fig. 2G-H). To prevent off-target effects, we further constructed a circIGF2BP3-si-mut plasmid with a mutation in the sh-circIGF2BP3-targeted back-splicing junction (Fig. S2E). Ectopic expression of circIGF2BP3-si-mut in NSCLC cells reversed the immune-promoting effects of si-circIGF2BP3 (Fig. S2F). Moreover, by silencing IGF2BP3 mRNA through siRNA-targeting exons that did not exist in circIGF2BP3, we found that the effect of circIGF2BP3 was independent of linear IGF2BP3 mRNA (Fig. S2G).

To clarify whether the immunosuppressive effect of circIGF2BP3 was mediated by CD8^+^ T cells, we inoculated circIGF2BP3-overexpressing LLC cells into immunocompetent C57BL/6 mice with or without administration of CD8 mAb, as previously described [30] (Fig. 2I, Fig. S2H). Compared with IgG2b treatment, CD8 mAb administration markedly enhanced tumor growth, decreased CD8^+^ T cell infiltration, and suppressed the cytotoxic effect of T cells (as indicated by GzmB^+^ CD8^+^, TNF-α^+^ CD8^+^, perforin^+^ CD8^+^ and IFN-γ^+^ CD8^+^ T cells) in CTRL LLC cell-inoculated mice, confirming that CD8^+^ T cells are pivotal for antitumor immunity maintenance (Fig. 2J-M). circIGF2BP3 overexpression in LLC cells significantly increased the tumor burden and decreased the number of CD8^+^ TILs and inactivated T cells. In mice with CD8^+^ T cell depletion, the tumor burden and density of activated CD8^+^ T cells did not change significantly in the circIGF2BP3-OE group compared with the CTRL group, indicating that the immunosuppressive effect of circIGF2BP3 is dependent on inhibiting CD8^+^ T cells (Fig. 2J-M).

We next explored whether circIGF2BP3 was involved in the regulation of chemokine circuitries and other immune cell trafficking in the TME. We found that the level of C-C motif chemokine ligand 5 (CCL5), a chemokine critical for CD8^+^ T cell homing in tumor regions [31], was significantly decreased in tumors with circIGF2BP3 overexpression, as determined by qPCR (Fig. S3A). Examining the density of Tregs (CD4^+^ Foxp3^+^), M2-like macrophages (CD68^+^ CD206^+^), and MDSCs (CD11b^+^) that infiltrated implanted tumors showed that circIGF2BP3 did not impact the recruitment of Tregs, TAMs or MDSCs in the TME (Fig. S3B-D). Taken together, these results strongly indicate that the tumor-promoting roles of circIGF2BP3 are dependent upon inactivating CD8^+^ T cells.

### Elevated circIGF2BP3 expression in NSCLC is due to m^6^A RNA methylation by METTL3

**Fig. 3:**
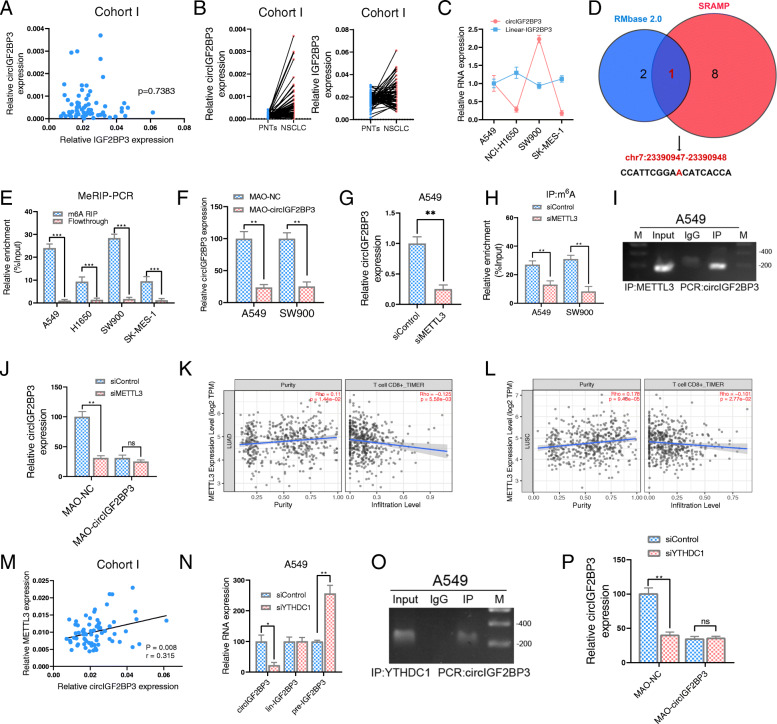
METTL3-mediated m^6^A modification promotes back-splicing of circIGF2BP3. **A** Correlation between circIGF2BP3 expression and linear mRNA expression in 68 paired samples of NSCLC in cohort I from Tongji Hospital. **B** The relative expression of circIGF2BP3 (left) and linear IGF2BP3 (right) in 68 paired samples of NSCLC in cohort I from Tongji Hospital. GAPDH was used as the internal control. **C** The relative expression of circIGF2BP3 and linear IGF2BP3 in 4 NSCLC cell lines. GAPDH was used as the internal control. **D** Venn diagram showing candidate m^6^A sites in circIGF2BP3 that were predicted by both SRAMP and RMBase. **E** MeRIP-qPCR analysis of the abundance of m^6^A-modified circIGF2BP3 in four NSCLC cell lines. **F** The relative expression of circIGF2BP3 in A549 and SW900 cells after treatment with MAO-circIGF2BP3 or MAO-NC, as detected by qRT-PCR. **G** The relative expression of circIGF2BP3 in A549 cells after treatment with siMETTL3 or siControl, as detected by qRT-PCR. **H** MeRIP-qPCR analysis of the relative abundance of m^6^A-modified circIGF2BP3 in NSCLC cells treated with siMETTL3 or siControl. **I** METTL3 IP assay to detect circIGF2BP3 in A549 cells. **J** The relative expression of circIGF2BP3 in siMETTL3 or siControl A549 cells after treatment with MAO-circIGF2BP3 or MAO-NC, as detected by qRT-PCR**. K-L** Correlation between METTL3 expression and tumor purity (left) and the infiltration level of CD8^+^ T cells (right) in LUSC (**K**) and LUAD (**L**) from a TCGA data set analyzed by the TIMER algorithm. **M** Correlation between circIGF2BP3 and METTL3 expression in 68 paired samples of NSCLC in cohort I from Tongji Hospital. GAPDH was used as the internal control. **N** The relative expression of circIGF2BP3, linear IGF2BP3, and IGF2BP3 pre-RNA in A549 cells treated with siControl or siYTHDC1, as measured by qRT-PCR. **O** YTHDC1 IP assay to detect circIGF2BP3 in A549 cells. **P** The relative expression of circIGF2BP3 in siYTHDC1 or siControl A549 cells after treatment with MAO-circIGF2BP3 or MAO-NC, as detected by qRT-PCR**.** Data represent the mean ± SD. *P < 0.05, **P < 0.01, ***P < 0.001. *P* values were determined by unpaired Student’s t test (**E, F, G, H, J, N** and **P**). Correlations were determined by the Pearson (**A** and **M**) or Spearman correlation test (**K** and **L**)

Exon-derived circRNAs and their host linear mRNAs are derived from the same pre-mRNA. IGF2BP3 is reportedly overexpressed in NSCLC [32]; thus, we investigated whether circIGF2BP3 overexpression was due to the increased transcription of its host gene. For this, we analyzed the coexpression relationship between circIGF2BP3 and IGF2BP3. The circIGF2BP3 level was not correlated with the linear IGF2BP3 level (Fig. 3A), and the change in circIGF2BP3 expression in paired NSCLC tissues was much greater than that in IGF2BP3 tissues (Fig. 3B). Moreover, although IGF2BP3 expression was comparable across the four NSCLC cell lines, the circIGF2BP3 expression patterns were significantly different (Fig. 3C). Therefore, high expression of IGF2BP3 in NSCLC might not account for circIGF2BP3 upregulation, and the detailed mechanism remains elusive.

m^6^A modification plays a pivotal role in posttranscriptional regulation and the biogenesis of circRNAs [33]. To test whether circIGF2BP3 is modulated by m^6^A, we used SRAMP and RMBase 2.0 to predict potential m^6^A sites in circIGF2BP3 and found one m^6^A site shared by the two programs (Fig. 3D, Fig. S4A-B). Moreover, the MeRIP results demonstrated that circIGF2BP3 was more densely m^6^A modulated in NSCLC cells with high circIGF2BP3 expression than in those with low circIGF2BP3 expression (Fig. 3E). By using sequence-specific morpholino antisense oligos (MAOs) targeting the m^6^A site of circIGF2BP3, we found that NSCLC cells treated with MAO targeting circIGF2BP3 (MAO-circIGF2BP3) had drastically suppressed circIGF2BP3 expression and decreased m^6^A levels of circIGF2BP3 compared with cells treated with control MAO (MAO-NC) (Fig. 3F, Fig. S4C). We further constructed circIGF2BP3 with a mutation in the m^6^A site (termed circIGF2BP3-m^6^A-mut), and MeRIP-qPCR confirmed that circIGF2BP3-m^6^A-mut exhibited lower m^6^A levels than WT circIGF2BP3 (Fig. S4D-E). These data indicated the potential importance of m^6^A modification for circIGF2BP3 production.

The aberrant expression of m^6^A writers (METTL3, METTL14 and WATP) reportedly contributes to the malignant progression of NSCLC [34]. Thus, we silenced m^6^A writers separately and found that METTL3 knockdown, but not METTL14 or WTAP knockdown, decreased circIGF2BP3 expression (Fig. 3G, Fig. S4F-I). In addition, although METTL3 silencing inhibited m^6^A modification of WT circIGF2BP3 (Fig. 3H), the abundance of circIGF2BP3-m^6^A-mut and the levels of m^6^A modification in circIGF2BP3-m^6^A-mut were not altered by METTL3 silencing (Fig. S4J-K). RIP assays further validated the interaction between circIGF2BP3 and METTL3 (Fig. 3I). Notably, MAO-circIGF2BP3 treatment blunted the regulatory effect of METTL3 on circIGF2BP3 expression (Fig. 3J). The analysis of TILs by TIMER indicated that METTL3 negatively regulated CD8^+^ T cell infiltration (Fig. 3K-L). Meanwhile, the expression of circIGF2BP3 significantly correlated with METTL3 expression in cohort I (Fig. 3M). Together, these results confirmed that m^6^A modification affected the expression of circIGF2BP3 and required METTL3.

YTH domain containing 1 (YTHDC1) has been shown to be an m^6^A reader that regulates circRNA back-splicing [35]. Consistent with this finding, we found that YTHDC1 silencing in A549 cells significantly decreased circIGF2BP3 expression, paralleled by an increase in its precursor transcript, whereas the linear IGF2BP3 mRNA remained unchanged (Fig. 3N, Fig. S4L-M), suggesting that YTHDC1 might regulate m^6^A-mediated circIGF2BP3 biogenesis by promoting its back-splicing. RIP confirmed that circIGF2BP3 is an exogenous binding partner of YTHDC1 (Fig. 3O). m^6^A blockade by MAO-circIGF2BP3 almost completely abrogated the positive effect of YTHDC1 on circIGF2BP3 expression (Fig. 3P). Moreover, the subcellular localization and RNA stability of circIGF2BP3 were not altered by YTHDC1 depletion, as determined by subcellular qRT-PCR and actinomycin D assay, respectively, ruling out the possibility that YTHDC1 affects the nuclear retention or stability of circRNA (Fig. S4N-O). Taken together, our data suggest that the METTL3-mediated m^6^A methylation of circIGF2BP3 is responsible for circIGF2BP3 upregulation by promoting its back-splicing and circularization via YTHDC1.

### circIGF2BP3 suppresses antitumor immunity in NSCLC by elevating PKP3 expression

**Fig. 4:**
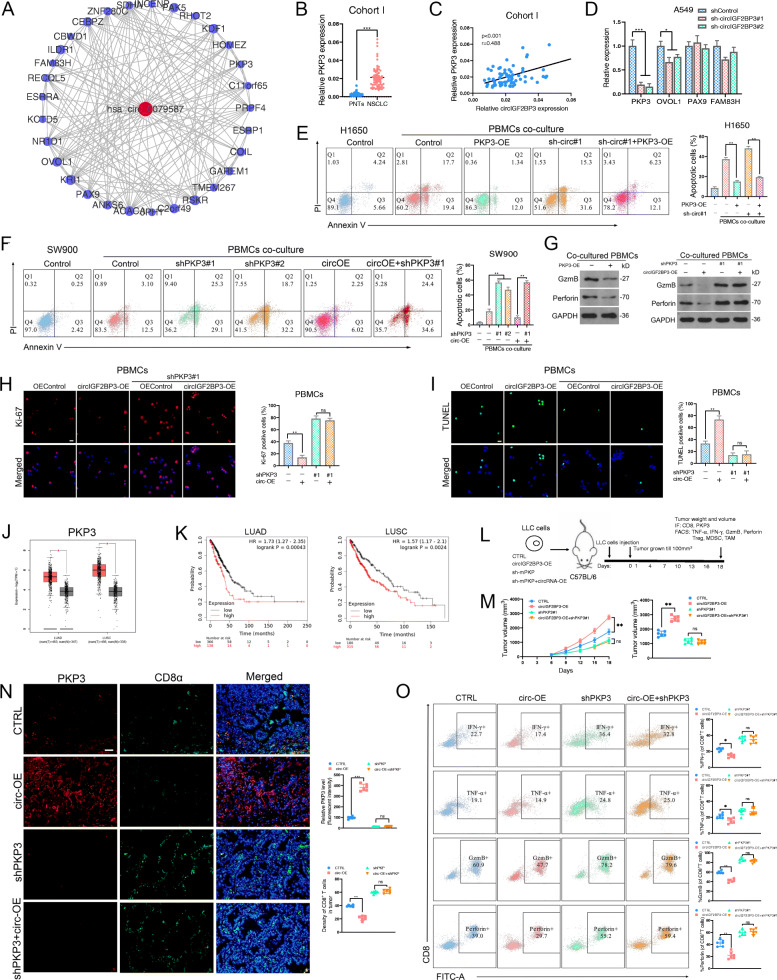
circIGF2BP3 attenuates antitumor immunity by upregulating PKP3 expression. **A** Coexpression network containing circIGF2BP3 and its 24 associated mRNAs built by Cytoscape. **B** The relative expression of PKP3 in 68 paired samples of NSCLC in cohort I from Tongji Hospital. GAPDH was used as the internal control. **C** Correlation between circIGF2BP3 and PKP3 expression in 68 paired samples of NSCLC in cohort I. **D** The relative expression of PKP3, OVOL1, PAX9 and FAM83H in A549 cells transfected with shControl, sh-circIGF2BP3#1 or sh-circIGF2BP3#2, as determined by qRT-PCR. **E-F** Representative images (left) and statistical quantitation (right) of the FACS analysis of the PBMC-mediated elimination of NSCLC cells, as determined by annexin V-FITC and propidium iodide (PI) double labeling. **G** The protein levels of perforin and GzmB in activated PBMCs after coculturing with the indicated NSCLC cells were detected by western blotting. **H-I** Ki-67 (**H**) and TUNEL (**I**) staining of PBMCs after coculturing with the indicated NSCLC cells. Scale bars, 20 μm. **J** The normalized expression of PKP3 in LUAD (left) and LUSC (right) from a TCGA data set. **K** K-M plot showing the relationship between circIGF2BP3 levels and the OS of patients with LUAD (left) and LUSC (right) in a TCGA data set. L Schematic diagram exhibiting the grouping and treatment plan of the in vivo study: C57BL/6 mice were inoculated with 10^6^ CTRL, circIGF2BP3-OE, sh-mPKP3 or sh-mPKP3 + circIGF2BP3-OE LLC cells, and the tumor volume was measured at the indicated time points. **M** Time-course evaluation of the tumor volumes of different groups measured every 3 days. **N** Representative images and statistical quantitation of IF CD8α and PKP3 staining in the indicated groups. Scale bars, 100 μm. **O** Representative images and statistical quantitation of the FACS analysis of IFN-γ^+^, TNF-α^+^, GzmB^+^ and perforin^+^ CD8^+^ TILs from the indicated xenograft tumor samples. Data represent the mean ± SD. *P < 0.05, **P < 0.01, ***P < 0.001. *P* values were determined by unpaired Student’s t test (**J**), paired Student’s t test (**B**), one-way ANOVA with Tukey’s post hoc test (**D, E, F, H, I, M, N** and **O**) and the log-rank test (**K**). Correlations were determined by the Pearson correlation test (**C**)

By analyzing the coexpression patterns of circIGF2BP3 and mRNAs from GSE126533, we identified 24 mRNAs that coexpressed with circIGF2BP3 (Pearson correlation coefficient ≥ 0.95, Fig. 4A). Among these, PAX9, OVOL1, FAM83H and PKP3 were further selected as putative mRNAs targeted by circIGF2BP3 based on ceRNA network analysis (Fig. 1F). Validation via qRT-PCR demonstrated that compared with PAX9, OVOL1 and FAM83H, PKP3 exhibited the most significant differential expression in 68 paired NSCLC samples (Fig. 4B, Fig. S5A). Moreover, the coexpression analyses identified PKP3 as the most statistically significant mRNA coexpressed with circIGF2BP3 (Fig. 4C, Fig. S5B). Although OVOL1 might also be regulated by circIGF2BP3, this effect was much weaker than that of PKP3 (Fig. 4D). For PAX9, although circIGF2BP3 was correlated with PAX9, no significant change in PAX9 expression was observed following circIGF2BP3 silencing (Fig. 4D, Fig. S5B). The upregulation of FAM83H expression in NSCLC samples was limited (Fig. S5A). Furthermore, circIGF2BP3 overexpression strongly increased PKP3 expression (Fig. S5C). Based on these observations, we regard PKP3 as a major candidate target of circIGF2BP3.

To explore whether the immunosuppressive effect of circIGF2BP3 was mediated via PKP3, we generated shPKP3 and PKP3-OE cell lines (Fig. S5D-E). An in vitro T cell-mediated killing assay showed that PKP3 silencing sensitized NSCLC cells to PBMC-mediated cytolysis and enhanced the expression of cytotoxicity-associated molecules in cocultured PBMCs, whereas PKP3 overexpression exhibited the opposite effect (Fig. 4E-G, Fig. S5F). Moreover, PKP3 overexpression significantly enhanced the immunosuppressive effects of NSCLC cells, while PKP3 knockdown abrogated circIGF2BP3-mediated effects (Fig. 4H-I, Fig. S5H). Moreover, PKP3 was exclusively expressed in NSCLC cells and not in immune cells isolated from PBMCs (Fig. S5G). The analysis of NSCLC patients in the TCGA data set confirmed the increased PKP3 expression in tumors compared with normal tissues (Fig. 4J). Survival analysis using the TCGA data also indicated that higher PKP3 expression correlated with worse OS (Fig. 4K).

To further validate the functional role of PKP3 in circIGF2BP3-mediated immune escape in vivo, we inoculated syngeneic C57BL/6 mice with circIGF2BP3-OE alone, circIGF2BP3-OE + shPKP3, shPKP3 alone or CTRL LLC cells (Fig. 4L). circIGF2BP3 overexpression upregulated mouse PKP3 expression in LLC cells, indicating the existence of the circIGF2BP3/PKP3 axis in mice (Fig. 4N). Furthermore, PKP3 silencing significantly blunted circIGF2BP3 overexpression-mediated immunosuppressive effects, as determined by assessing the tumor volume (Fig. 4M). IF staining and FACS analysis showed that PKP3 silencing was sufficient to abrogate the circIGF2BP3 overexpression-elicited decrease in the CD8^+^ TIL population and suppressed activated CD8^+^ T cells in the tumor region (Fig. 4N-O). Consistent with the results in Fig. S3, neither circIGF2BP3 nor PKP3 influenced the infiltration of Tregs, TAMs or MDSCs in the tumor region (Fig. S5J). Moreover, circIGF2BP3-mediated CCL5 downregulation in the tumor region was blocked following PKP3 silencing (Fig. S5I). Correlation analysis also confirmed the negative relationship between PKP3 and CCL5 expression in the NSCLC TCGA data set (Fig. S5K). qPCR analysis revealed that PKP3 expression in infiltrated CD8^+^ T cells was almost absent compared with that in LLC cells, indicating that the circIGF2BP3/PKP3 regulatory axis functions mainly in tumor cells (Fig. S5L). Taken together, these data confirmed that circIGF2BP3 promotes tumor immune escape by upregulating PKP3 expression in NSCLC cells.

### circIGF2BP3 upregulates PKP3 expression by sponging miR-328-3p and miR-3173-5p

**Fig. 5:**
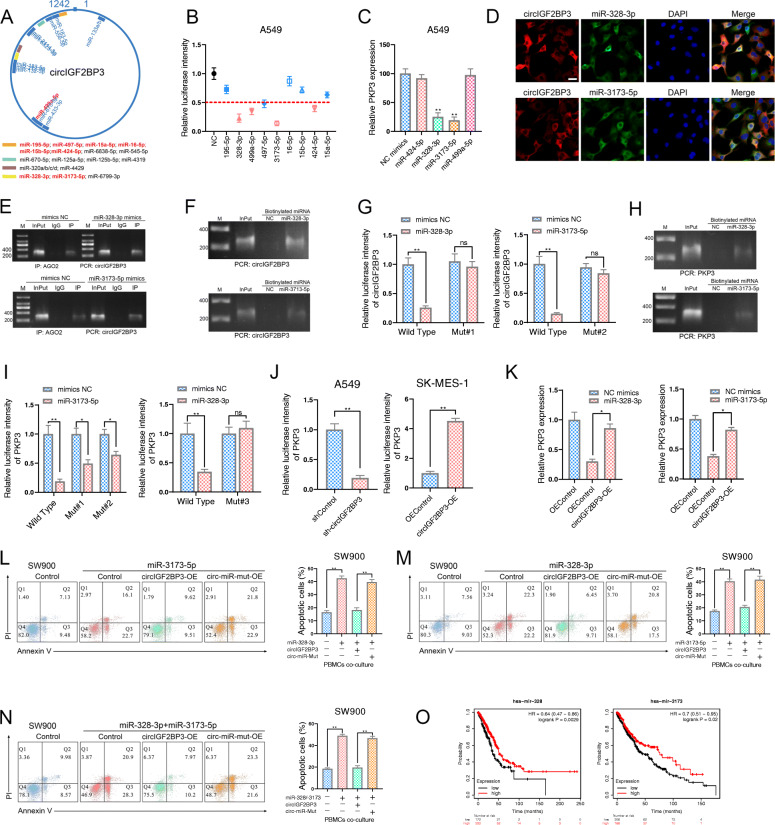
circIGF2BP3 elevates PKP3 expression by acting as an miRNA sponge for miR-328-3p and miR-3173-5p. **A** Schematic diagram illustrating the binding sites of circIGF2BP3 and its predicted miRNAs. miRNAs marked in red denote miRNAs containing binding sites for both circIGF2BP3 and PKP3, as predicted by starBase. **B** The relative luciferase activity of the circIGF2BP3 reporter in A549 cells transfected with the indicated miRNA mimics. Firefly luciferase activity was normalized to Renilla luciferase activity. **C** The relative expression of PKP3 in A549 cells transfected with the indicated miRNA mimics was detected by qRT-PCR. **D** FISH shows the colocalization of circIGF2BP3 with miR-328-3p and miR-3173-5p. circIGF2BP3 FISH (red), miR-328-3p FISH (green), miR-3173-5p FISH (green), nuclear DAPI staining (blue). Scale bars, 20 μm. **E** AGO2 IP assay to detect circIGF2BP3 in A549 cells transfected with miR-328-3p mimics (upper) or miR-3173-5p mimics (lower). **F** PCR detection of circIGF2BP3 enriched with 3′-end biotinylated miR-328-3p or 3′-end biotinylated miR-3173-5p. **G** The relative luciferase activity of the circIGF2BP3 reporter and its mutant in A549 cells transfected with miR-328-3p mimics (left) or miR-3173-5p mimics (right). Firefly luciferase activity was normalized to Renilla activity. **H** PCR detection of PKP3 enriched with 3′-end biotinylated miR-328-3p (upper) or 3′-end biotinylated miR-3173-5p (lower). **I** The relative luciferase activity of the PKP3–3’UTR reporter and its mutant in A549 cells transfected with miR-3173-5p mimics (left) or miR-328-3p mimics (right). Firefly luciferase activity was normalized to Renilla activity. **J** The relative luciferase activity of the PKP3–3’UTR reporter in circIGF2BP3-silenced (left) and circIGF2BP3-overexpressing (right) NSCLC cells. **K** The relative expression of PKP3 in A549 cells transfected with the indicated miRNA mimics alone or cotransfected with circIGF2BP3. **L-N** FACS analysis of PBMC-mediated elimination of NSCLC cells transfected with the indicated constructs after treatment with miR-328-3p mimics (**L**), miR-3173-5p mimics (**M**), or miR-328-3p and miR-3173-5p mimics (**N**). **O.** K-M OS analysis of NSCLC patients in the TCGA data set according to the expression levels of miR-328-3p (left) and miR-3173-5p (right). Data represent the mean ± SD. *P < 0.05, **P < 0.01, ***P < 0.001. *P* values were determined by unpaired Student’s t test (**G, I** and **J**), one-way ANOVA with Tukey’s post hoc test (**B, C, K, L, M** and **N**) and the log-rank test (**O**)

circRNAs can act as ceRNAs to regulate gene expression [29]. Given that circIGF2BP3 is mainly located in the cytoplasm, we hypothesized that circIGF2BP3 acting as a miRNA sponge might be the mechanism responsible for PKP3 upregulation. As shown in Fig. 1F, we constructed a circIGF2BP3-miRNA-PKP3 network based on prediction by starBase. This network contained 9 miRNAs that shared common binding sites for circIGF2BP3 and PKP3 (Fig. 5A). By conducting a luciferase reporter assay, we found that 4 miRNAs could efficiently reduce the luciferase activity of the circIGF2BP3 reporter (Fig. 5B). Among these miRNAs, only the overexpression of miR-328-3p and miR-3173-5p decreased PKP3 levels (Fig. 5C). RNA-FISH confirmed the colocalization of circIGF2BP3 with miR-328-3p and miR-3713-5p (Fig. 5D). AGO2 RIP and biotinylated miRNA pulldown assays further validated the enrichment of circIGF2BP3 with miR-328-3p and miR-3713-5p (Fig. 5E-F). Moreover, the luciferase reporter assays we performed demonstrated that both miR-328-3p and miR-3713-5p failed to decrease the luciferase activity of the circIGF2BP3 reporter when the potential miRNA binding sites were mutated (Fig. 5G, Fig. S6A). These data suggest a role of circIGF2BP3 as a ceRNA in sponging miR-328-3p and miR-3713-5p in NSCLC cells.

We further confirmed that miR-328-3p and miR-3713-5p were able to decrease PKP3 expression by targeting its 3’UTR. First, biotin-coupled miRNA pulldown demonstrated the enrichment of miR-328-3p and miR-3173-5p with PKP3 (Fig. 5H). Second, the luciferase activity of the PKP3 3’UTR WT reporter was significantly reduced when the cells were transfected with miR-328-3p or miR-3173-5p compared with that of the mutated form of the PKP3 3’UTR (Fig. 5I, Fig. S6B). Third, circIGF2BP3 overexpression increased the luciferase activity of the PKP3 reporter, while circIGF2BP3 silencing decreased it (Fig. 5J). Finally, qPCR confirmed that miR-328-3p and miR-3173-5p alone or in combination significantly reduced the mRNA level of PKP3, but these effects were rescued by circIGF2BP3 overexpression (Fig. 5K, Fig. S6C). These data indicate that circIGF2BP3 upregulates PKP3 expression through competitive binding with miR-328-3p and miR-3173-5p.

We next investigated the effect of miR-328-3p and miR-3173-5p on NSCLC cell-mediated immune escape. The overexpression of miR-328-3p or miR-3173-5p promoted the cytotoxic effect of PBMCs, which was reversed by circIGF2BP3 overexpression (Fig. 5L-N). We further mutated the miR-328-3p and miR-3173-5p binding sites on circIGF2BP3, and rescue assays showed that circIGF2BP3-miR-mut influenced neither miR-328-3p- nor miR-3173-5p-mediated PBMC activation (Fig. 5L-N, Fig. S6D). Finally, survival analysis of the TCGA data set showed that NSCLC patients with lower miR-328-3p or miR-3173-5p expression had worse predicted OS (Fig. 5O).

Taken together, these results confirmed that circIGF2BP3 could specifically sponge miR-328-3p and miR-3173-5p, relieving their repression on PKP3 and thereby inducing a tumor immunosuppressive effect in NSCLC.

### PKP3 stabilizes PD-L1 by promoting the OTUB1-mediated deubiquitination of PD-L1

**Fig. 6:**
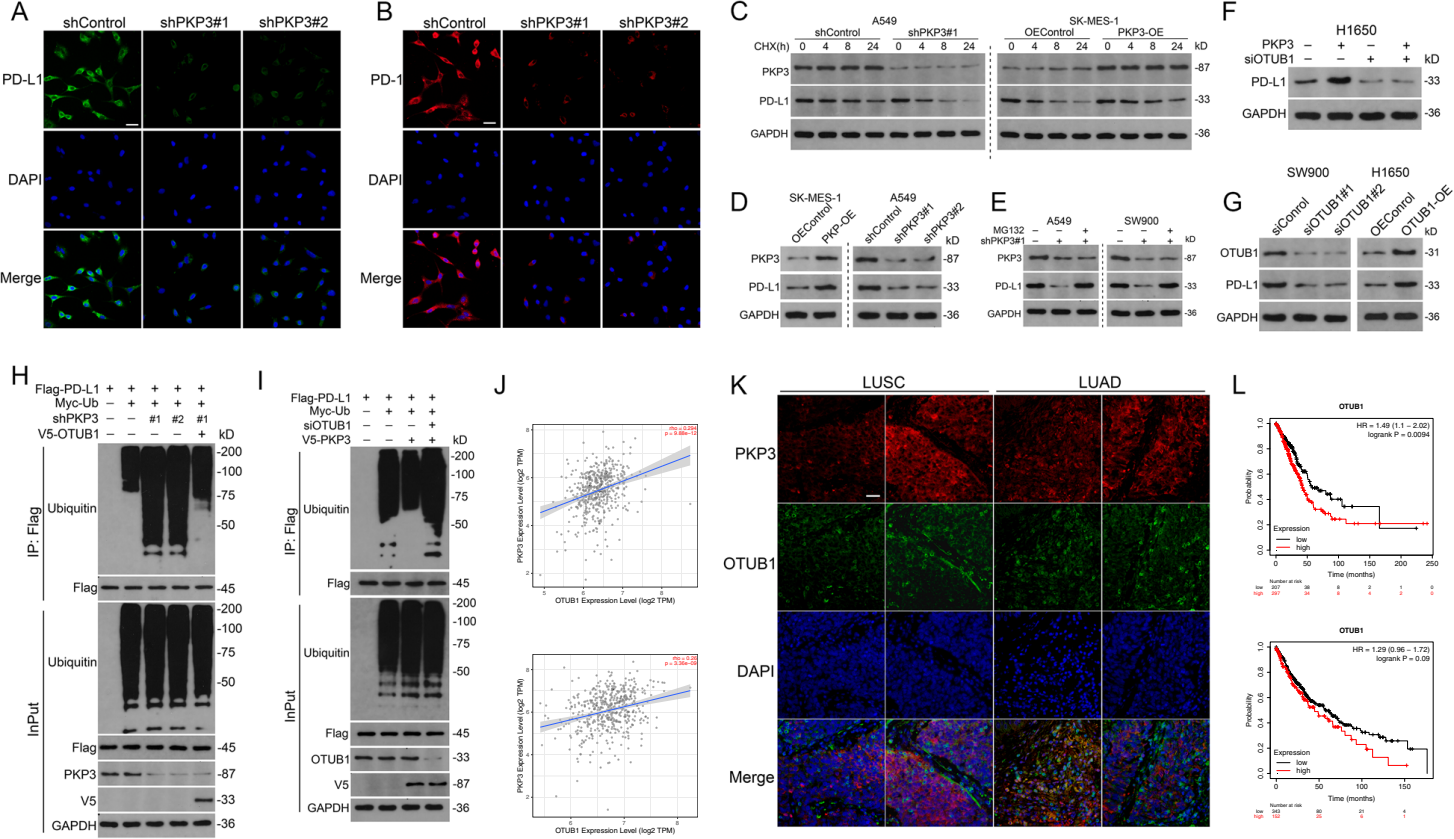
PKP3 stabilizes PD-L1 via OTUB1-mediated PD-L1 deubiquitination. **A** IF staining of PD-L1 in A549 cells treated with shControl or shPKP3. PD-L1 (green), nuclear DAPI staining (blue). Scale bars, 20 μm. **B** IF staining to detect PD-1 binding ability. PD-L1 in A549 cells treated with shControl or shPKP3. PD-1 (red), nuclear DAPI staining (blue). Scale bars, 20 μm. **C** Western blotting analysis of PD-L1 stability in A549 cells expressing PKP3 shRNA and in SK-MES-1 cells transfected with PKP3-overexpressing plasmid. **D** The protein level of PD-L1 in PKP3-silenced or PKP3-overexpressing NSCLC cells was detected by western blotting. **E** Western blotting analysis of PD-L1 stability in NSCLC cells transfected with PKP3 shRNA after treatment with MG132 (100 μM). **F** Western blotting analysis of PD-L1 abundance in CTRL or PKP3-overexpressing H1650 cells treated with or without OTUB1 siRNA. **G** OTUB1 overexpression increased PD-L1 protein levels in NSCLC cells, whereas OTUB1 inhibition reduced these levels, as determined by western blotting. **H** IP analysis of PD-L1 ubiquitination in A549 cells transfected with the indicated constructs and/or PKP3 shRNA. **I** IP analysis of PD-L1 ubiquitination in A549 cells transfected with the indicated constructs and/or OTUB1 siRNA. **J** Correlation between PKP3 and OTUB1 mRNA levels in LUAD (upper) and LUSC (lower) from the TCGA data set, as analyzed by TIMER 2.0. **K** Representative FISH-IF images of PKP3 protein and OTUB1 mRNA in NSCLC samples from Tongji Hospital. Scale bars, 100 μm. **L** K-M OS analysis of NSCLC patients in the TCGA data set according to the OTUB1 expression level. Data represent the mean ± SD. *P < 0.05, **P < 0.01, ***P < 0.001. *P* values were determined by the log-rank test (**L**). Correlations were determined by the Spearman correlation test (**J**)

Hyperactivation of PD-L1 in tumor cells strongly inhibits T cell activation and accounts for cancer cell escape from immune surveillance [36]. We thus tested whether circIGF2BP3/PKP3 axis-mediated immune escape was mediated by PD-L1. Consistent with our prediction, PKP3 silencing significantly reduced the abundance of the PD-L1 protein and decreased PD-1 binding to the NSCLC cell surface (Fig. 6A-B). However, although PKP3 depletion decreased the protein level of PD-L1 (as determined by immunoblotting), PKP3 did not influence PD-L1 mRNA expression in NSCLC cells (Fig. 6D, Fig. S7A-B). Correlation analyses of the TCGA data set also corroborated this finding (Fig. S7C). This indicated that PKP3-mediated PD-L1 induction is possibly posttranscriptional but not transcriptional. Accordingly, a CHX chase assay revealed that PKP3 overexpression stabilized, while PKP3 silencing accelerated, the turnover of PD-L1 (Fig. 6C). Moreover, proteasome inhibition by MG132 significantly blocked PKP3 deficiency-induced proteolysis (Fig. 6E). Taken together, these data suggest that PKP3 might rescue PD-L1 from proteasomal degradation.

The potential mechanism by which PKP3 regulates the ubiquitination of PD-L1 was next investigated. NSCLC cells were transfected with siRNA targeting various E3 ligases or deubiquitinating enzymes reported to be involved in the ubiquitination and deubiquitination of PD-L1 [28]. We showed that silencing of only the deubiquitinating enzyme otubain-1 (OTUB1) completely abrogated PKP3 overexpression-mediated stabilization of the PD-L1 protein (Fig. 6F, Fig. S7D-I). In addition, OTUB1 overexpression increased, while OTUB1 silencing decreased, PD-L1 protein levels (Fig. 6G). Zhu et al. previously confirmed that OTUB1 acts as a deubiquitinase that stabilizes PD-L1 [36]. To corroborate this finding, we analyzed PD-L1 ubiquitination in NSCLC cells and demonstrated that PD-L1 ubiquitination was greatly enhanced in cells transfected with PKP3 shRNA compared with control cells or PKP3-silenced/OTUB1-overexpressing cells (Fig. 6H). In reciprocal experiments, PKP3 overexpression significantly suppressed PD-L1 ubiquitination, which was completely reversed by OTUB1 silencing (Fig. 6I). Bioinformatics analysis of the TCGA database revealed that PKP3 expression was positively correlated with OTUB1 mRNA levels in both LUSC and LUAD cells (Fig. 6J). FISH and IF staining also confirmed the coexpression pattern of PKP3 protein and OTUB1 mRNA in NSCLC tissues (Fig. 6K). The survival analysis of the TCGA database showed that high OTUB1 expression predicts poor OS of NSCLC patients (Fig. 6L). Overall, PKP3 stabilizes the PD-L1 protein in an OTUB1-dependent manner.

### PKP3 upregulates OTUB1 expression by increasing OTUB1 mRNA stability

**Fig. 7:**
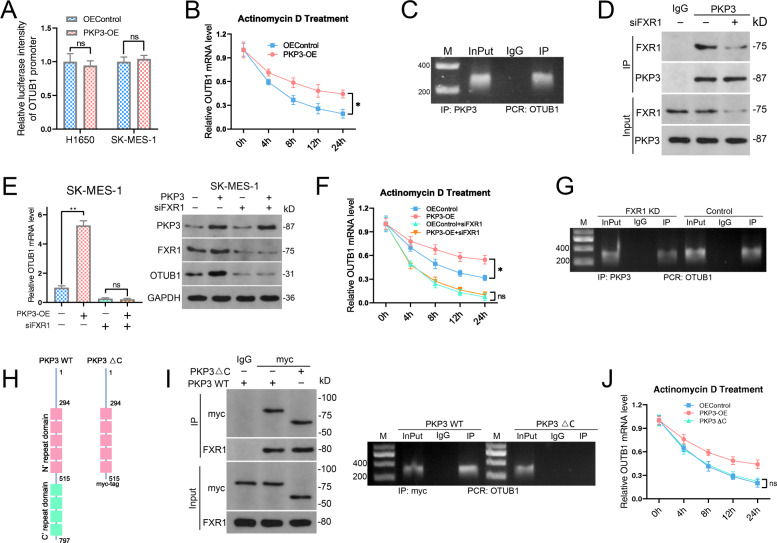
PKP3 promotes OTUB1 expression by enhancing its mRNA stability. **A** Relative luciferase activity of the OTUB1 promoter in NSCLC cells transfected with PKP3-overexpressing constructs. Firefly luciferase activity was normalized to Renilla activity. **B** Time-course qRT-PCR analyses of the relative abundance of OTUB1 in control or PKP3-overexpressing H1650 cells treated with actinomycin D (10 μg/ml). **C** PKP3 IP assay to detect OTUB1 mRNA in A549 cells. **D** IP analysis of the interaction between FXR1 and PKP3 in A549 cells. **E** The expression of OTUB1 in FXR1-silenced SK-MES-1 cells with or without PKP3 overexpression, as determined by qRT-PCR (left) and western blotting (right). **F** Time-course qRT-PCR analyses of the relative abundance of OTUB1 mRNA in FXR1-silenced SK-MES-1 cells with or without PKP3 overexpression after treatment with actinomycin D (10 μg/ml). **G** PKP3 IP assay to detect OTUB1 in A549 cells with or without FXR1 knockdown. **H** Schematic diagram showing the sequence of myc-tagged full-length PKP3 and myc-tagged PKP3ΔC lacking repeat domains at its C-terminus. **I** The interaction between PKP3 constructs (full-length PKP3 or PKP3ΔC) and OTUB1 mRNA was analyzed by qRT-PCR after IP. The enrichment of myc-tagged proteins and coprecipitated FXR1 were also analyzed. **J** Time-course qRT-PCR analyses of the relative abundance of OTUB1 in H1650 cells transfected with full-length PKP3 or PKP3ΔC after treatment with actinomycin D (10 μg/ml). Data represent the mean ± SD. *P < 0.05, **P < 0.01, ***P < 0.001. *P* values were determined by unpaired Student’s t test (**A** and **B**) and one-way ANOVA with Tukey’s post hoc test (**E, F** and **J**)

To test whether PKP3-mediated OTUB1 upregulation is posttranscriptional or transcriptional, we constructed and transfected a luciferase reporter plasmid containing a promoter fragment of OTUB1 into NSCLC cells stably overexpressing PKP3. We found similar luciferase activity in PKP3-overexpressing cells and control cells (Fig. 7A). Moreover, PKP3 markedly prevented OTUB1 mRNA degradation (Fig. 7B). Taken together, these findings indicate that PKP3 upregulates OTUB1 expression at the posttranscriptional level rather than at the transcriptional level, possibly by increasing the stability of OTUB1 mRNA.

PKP3 reportedly increases mRNA stability by associating with fragile X mental retardation syndrome-related protein 1 (FXR1) [22]. To test whether PKP3 also regulates OTUB1 expression in this manner, we first analyzed PKP3-bound RNA by RIP. The results showed that PKP3 could interact with OTUB1 mRNA (Fig. 7C). PKP3 forms a complex with FXR1, and recruitment of RNA into the PKP3-containing complex requires FXR1 [22]. Consistent with this, we found by IP that endogenous PKP3 could bind with FXR1 in A549 cells (Fig. 7D). PKP3 overexpression-mediated OTUB1 upregulation was completely blocked after FXR1 silencing, and we obtained similar results with actinomycin treatment (Fig. 7E-F). Ultimately, RIP showed that the interaction between PKP3 and OTUB1 mRNA requires FXR1 (Fig. 7G), and PKP3 lacking C-terminal arm repeats was unable to bind OTUB1 mRNA or maintain the mRNA stability (Fig. 7H-J).

Overall, we provide evidence that PKP3 is a major regulator of OTUB1 mRNA expression in an FXR1-dependent manner.

### PKP3 inhibition augments the efficacy of anti-PD-1 therapy in an immunocompetent mouse model

**Fig. 8:**
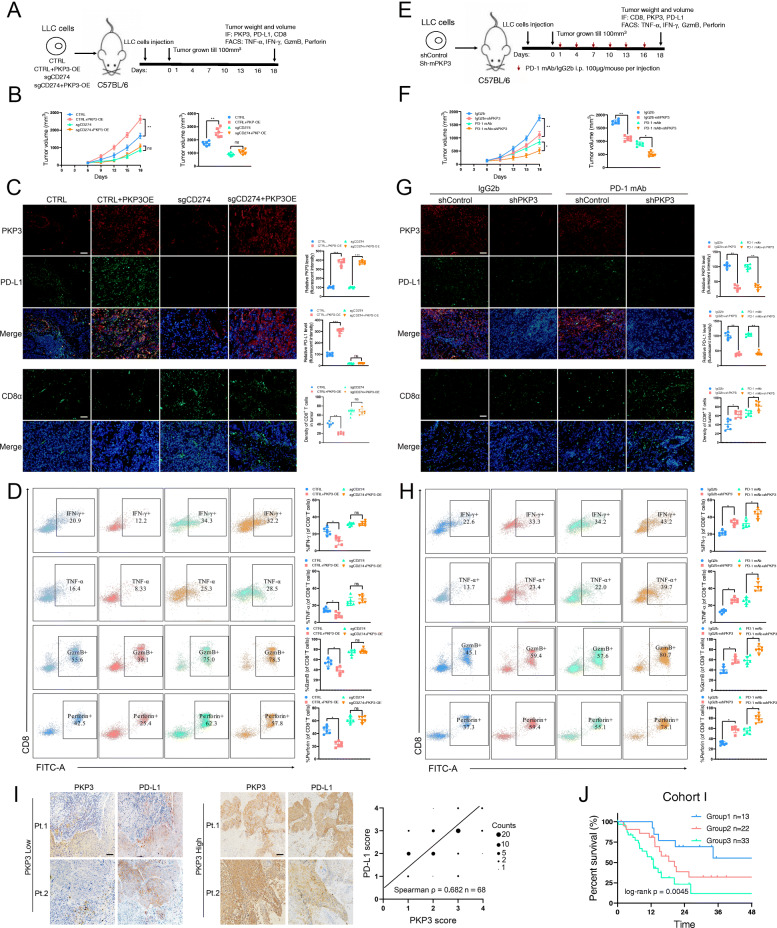
PKP3 suppresses antitumor immunity by regulating PD-L1, and PKP3/PD-L1 axis inhibition enhances the efficacy of anti-PD-1 therapy. **A** Schematic diagram showing the grouping and treatment plan of the in vivo study: C57BL/6 mice were inoculated with 10^6^ CTRL, CTRL+PKP3-OE, sgCD274 or sgCD274 + PKP3-OE LLC cells, and the tumor volume was measured at the indicated time points. **B** Time-course evaluation of tumor volumes measured every 3 days. **C** Representative images and statistical quantitation of IF staining of PKP3, PD-L1 and CD8α in the indicated groups. Scale bars, 100 μm. **D** Representative images and statistical quantitation of the FACS analysis of IFN-γ^+^, TNF-α^+^, GzmB^+^ and perforin^+^ CD8^+^ TILs from the indicated xenograft tumor samples. **E** Schematic diagram showing the grouping and treatment plan of the in vivo study: C57BL/6 mice were inoculated with 10^6^ shControl or sh-mouse PKP3 LLC cells and received PD-1 mAb treatment or IgG2b control at the indicated time points. **F** Time-course evaluation of tumor volumes measured every 3 days. **G** Representative images and statistical quantitation of IF staining of PKP3, PD-L1 and CD8α in the indicated groups. Scale bars, 100 μm. **H** Representative images and statistical quantitation of the FACS analysis of IFN-γ^+^, TNF-α^+^, GzmB^+^ and perforin^+^ CD8^+^ TILs in the indicated xenograft tumor samples. **I** Representative images and statistical quantitation of IHC staining of PKP3 and PD-L1 in NSCLC samples from cohort I. Scale bars, 200 μm. **J** K–M analysis of OS of patients in cohort I. Patients were divided into group 1 (low PKP3/high TILs, *n* = 13), group 2 (low PKP3/low TILs or high PKP3/high TILs, *n* = 22) and group 3 (high PKP3/low TILs, *n* = 33). Data represent the mean ± SD. *P < 0.05, **P < 0.01, ***P < 0.001. *P* values were determined by one-way ANOVA with Tukey’s post hoc test (**B, C, D, E, F, G** and **H**) or the log-rank test (**J**) Correlations were determined by the Spearman correlation test (**I**)

To validate PKP3-mediated PD-L1 regulation in vivo, we constructed sgCD274 LLC cells using CRISPR-Cas9 (Fig. S7J) and inoculated sgCD274 LLC cells overexpressing PKP3 into C57BL/6 immunocompetent mice (Fig. 8A). The sgCD274 tumors exhibited significantly reduced volumes and decreased PD-L1 expression compared with CTRL tumors (Fig. 8B-C). Moreover, although PKP3 overexpression significantly inhibited CD8^+^ TIL infiltration and boosted tumor growth and PD-L1 expression in the CTRL group, this effect was significantly blocked in the sgCD274 group (Fig. 8C-D). Thus, our data indicated that the PKP3-mediated effect is dependent on tumor PD-L1.

We further tested the combinatory effect of PKP3 inhibition and PD-1 blockade in vivo. PKP3 was knocked down in C57BL/6 immunocompetent mice harboring LLC cells with or without anti-PD-1 treatment (Fig. 8E). The combination of PKP3 silencing and anti-PD-1 treatment led to a significant reduction in tumor burden compared with PKP3 knockdown or anti-PD-1 treatment alone (Fig. 8F). Moreover, immune profiling confirmed that the combination of PKP3 inhibition and PD-1 blockade significantly promoted the infiltration and activation of CD8^+^ T cells in the tumor region compared with that in any other group (Fig. 8G-H).

Diverse types of cells residing in the TME express PD-L1, which could suppress antitumor immunity in the absence of tumor cell PD-L1 expression. To explore the impact of PD-L1-expressing stromal cells on the treatment efficacy of combinatory PKP3 silencing and anti-PD-1 treatment, PKP3 knockdown LLC cells were implanted into PD-L1 WT and KO mice, which were then treated with anti-PD-1 antibody. We found that PD-1 blockage suppressed tumor growth and increased intratumoral CD8^+^ T cells in PD-L1 WT mice, whereas anti-PD-1 treatment had no detectable effect on tumor growth or CD8^+^ T cell infiltration in mice with PD-L1 deficiency in the host (Fig. S8A-D), indicating that PD-L1 expression on stromal cells also plays a role in regulating the efficacy of anti-PD-1 therapy. Moreover, PD-L1 overexpression resulting from either PKP3 overexpression or circIGF2BP3 overexpression was sufficient to impair anti-PD-1 treatment-mediated activated CD8^+^ T cell recruitment and tumor regression, also confirming the fact that circIGF2BP3/PKP3-mediated PD-L1 upregulation plays the main role in regulating anti-PD-1 responsiveness (Fig. S8E-G).

We next validated our findings in clinical samples. First, IHC staining showed that PKP3 expression was positively correlated with PD-L1 expression (Fig. 8I). Moreover, we stratified patients from cohort I according to PKP3 abundance and the number of CD8^+^ TILs in the tumor region into three groups (group 1: low PKP3/high TILs; group 2: low PKP3/low TILs or high PKP3/high TILs; and group 3: high PKP3/low TILs). Our survival analysis revealed that group 1 had the most favorable survival outcome, followed by group 2 and group 3, the latter of which had the shortest OS compared with the other groups (Fig. 8J). All of these results were consistent with our in vitro and in vivo experiments.

Taken together, our data demonstrated that combining PKP3 inhibition and PD-1 blockade may constitute a promising strategy to enhance the efficacy of anti-PD-1 therapy.

## Discussion

**Fig. 9:**
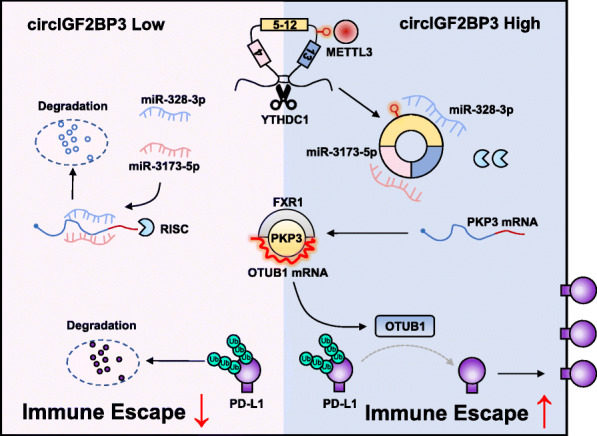
Model of circIGF2BP3-mediated immunosuppressive effect in NSCLC. circIGF2BP3 acts as a sponge for miR-328-3p and miR-3173-5p and elevates PKP3 expression, which stabilizes OTUB1 mRNA in an FXR1-dependent manner. OTUB1 removes ubiquitin chains from PD-L1 and subsequently hinders its degradation. circIGF2BP3-induced PD-L1 upregulation in NSCLC cells ultimately promotes immune evasion and resistance to anti-PD-1 therapy

In the present study, by employing WGCNA and immune infiltrate estimation implemented by CIBERSORT and TIMER, we identified candidate circRNAs that impact antitumor immunity in NSCLC. For the first time, we identified circIGF2BP3 as a markedly upregulated circRNA that is negatively associated with CD8^+^ T cell infiltration. In vitro and in vivo studies confirmed that circIGF2BP3 compromises the CD8^+^ T cell response by competitively elevating PKP3 expression. The immunosuppressive effect of PKP3 was dependent upon inducing the OTUB1-mediated deubiquitination of PD-L1, thus protecting PD-L1 from proteasomal-mediated degradation. Stabilized PD-L1 accumulates on the membrane of NSCLC cells; this accumulation contributes to the inactivity and exhaustion of T cells through interactions with PD-1 loaded on T cell membranes. This circIGF2BP3/PXP3/PD-L1 axis ultimately contributes to tumor cell escape from immune surveillance (Fig. 9). A syngeneic mouse model showed that combining PD-1 mAb administration and PKP3 silencing enhances the treatment efficacy of ICBs in NSCLC. We characterized a regulatory mechanism of circRNA-mediated immune evasion in NSCLC and highlighted that circIGF2BP3 may act as a potential therapeutic target to increase the efficacy of PD-1 antibodies.

circRNAs are now recognized as widespread and highly conserved endogenous ncRNAs that are highly stable and participate in several important cancer-related processes [37]. Although immunotherapy has provided substantial benefits for advanced NSCLC patients, the overall response rate barely exceeds 40%, and immune evasion of cancer cells poses major obstacles for immunotherapy [38]. Accumulating evidence suggests that the presence of CD8^+^ TILs is a hallmark of the antitumor immune response [39]. In our study, we performed WGCNA to enrich circRNAs that were both upregulated in NSCLC tissues and negatively regulated the population of CD8^+^ TILs. We further constructed a coexpression network between these circRNAs and key genes that inhibited CD8^+^ TIL infiltration to identify potential circRNAs. It has been acknowledged that circRNAs primarily function as miRNA sponges to competitively upregulate the expression of miRNA-targeted genes [40]. Based on this theory, we constructed a ceRNA network by analyzing the miRNA target prediction database starBase, as illustrated in Fig. 1F. Among the molecules in this network, circIGF2BP3 was characterized as an authentic circRNA that inactivates the CD8^+^ T cell response in vivo and in vitro, indicating the pivotal impact of circIGF2BP3 on the immune evasion of NSCLC cells.

m^6^A modification, one of the major posttranscriptional modifications of eukaryotic RNA, participates in various aspects of RNA production, RNA stability and interactions with cancers [41]. Generally, m^6^A modification is mainly mediated by m^6^A writers (METTL3, METTL14 and WTAP), erasers (FTO and ALKBH5) and readers (YTHDF, YTHDC and HNRNPC) [42]. Although the role of m^6^A mRNA modification has been extensively studied, the molecular function of m^6^A regarding circRNA biogenesis remains elusive. Here, we demonstrated that m^6^A modification, which requires the combined action of METTL3 and YTHDC1, was responsible for circIGF2BP3 upregulation in NSCLC. We showed that the deposition of m^6^A in the exonic region of the pre-transcript of IGF2BP3 favors back-splicing rather than linear splicing. To our knowledge, this is the first report to provide insight into the regulatory mechanism of m^6^A modification-mediated circRNA upregulation in NSCLC. In NSCLC, METTL3 has been found to induce drug resistance and the metastasis of tumor cells by promoting YAP translocation [20]. METTL3-induced increases in lncRNA ABHD11-AS1 expression promotes the proliferation and Warburg effect of tumor cells [43]. However, little is known about whether METTL3 participates in tumor immunity in NSCLC. We found that METTL3 facilitates the immune evasion of NSCLC cells by promoting the circularization of circIGF2BP3, and immune infiltration analysis by TIMER further confirmed that METTL3 negatively regulates CD8^+^ T cell infiltration (Fig. 3K). Therefore, our study not only dissected the molecular mechanism of circIGF2BP3 upregulation but also revealed a novel immunosuppressive effect of METTL3 in NSCLC.

PKP3 is highly expressed in various tumors, such as breast cancer [44], malignant pleural mesothelioma [45] and ovarian cancer [21]. Here, we found that PKP3 expression was significantly increased in NSCLC and that high PKP3 levels inactivated T cells both in vitro and in vivo and correlated with an unfavorable prognosis of NSCLC patients. Importantly, the immunosuppressive effect of circIGF2BP3 was dependent on elevating PKP3 expression by competitively binding with miR-328-3p and miR-3173-5p. miR-328-3p has been reported to alleviate the proliferation, migration and radio-resistance of NSCLC cells [46, 47], suggesting its suppressive effect on NSCLC progression. However, the role of miR-3173-5p in NSCLC remains elusive. In this study, we determined that miR-328-3p and miR-3173-5p were sponged by circIGF2BP3, resulting in fewer miRNAs targeting PKP3 and thus suppressing tumor immunity. The protective effect of both miRNAs was further confirmed by the survival analysis of TCGA data (Fig. 5O). Our identification of the circIGF2BP3-miR-328-3p/miR-3173-5p/PKP3 ceRNA regulatory axis improves our understanding of the mechanism of immune escape by NSCLC cells.

The regulatory mechanism of PD-L1 expression, which is highly associated with the efficacy and response rate of anti-PD-1/PD-L1 immunotherapy [48], merits further extensive investigation. It has been proven that PD-L1 can be regulated at both the transcriptional and posttranscriptional levels [30]. In this study, we showed that OTUB1, belonging to the OTU subfamily of deubiquitinases, functioned as a downstream effector of PKP3. OTUB1 deubiquitinated and thus prevented the degradation of PD-L1. Although PD-L1 has been reported to be ubiquitinated or deubiquitinated by CSN5, USP22, OTUB1, FBXO38, STUB1, SPOP or HRD1 [28], only OTUB1 silencing blocked the regulatory effect of PKP3 on PD-L1 expression (Fig. S6D-I, Fig. 6F-G). Apart from being a member of the desmosomal complex, cytoplasmic nonjunctional PKP3 can bind with the RNA-binding protein FXR1 to enhance the mRNA stability of desmoplakin and its family member PKP2 [22]. In line with this, we showed that PKP3 did not influence OTUB1 transcription but altered the rate of OTUB1 mRNA turnover, as demonstrated by actinomycin treatment (Fig. 7A-B). Co-IP and RIP further confirmed that the ability of PKP3 to stabilize OTUB1 mRNA required both its C-terminal arm repeat and FXR1 recruitment, while its C-terminal arm repeat was dispensable for the interaction between FXR1 and PKP3 (Fig. 7H-J), which is consistent with a previous study [22]. Therefore, our data revealed a novel mechanism that regulates PD-L1 expression.

In NSCLC patient samples, we identified a positive relationship between PKP3 and PD-L1 expression. A syngeneic mouse model further confirmed the inverse relationship between the circIGF2BP3/PKP3 axis and CD8^+^ T cell infiltration. Intriguingly, we found that the circIGF2BP3/PKP3 axis could also decrease the level of CCL5 in the TME, thus contributing to decreased CD8^+^ T cell recruitment into tumor regions. The role of the circIGF2BP3/PKP3 axis in shaping local chemokine networks requires further investigation. Finally, by assessing the clinical significance of the PKP3/PD-L1 axis and CD8^+^ T infiltration, we demonstrated that a combination of low PKP3 expression and high CD8^+^ T cell infiltration indicated a longer OS in NSCLC patients (Fig. 8J). Thus, we further identified a potential therapeutic target to alleviate tumor immune escape.

## Conclusion

In summary, our work reveals that circIGF2BP3 relieves the inhibitory effect of miR-328-3p and miR-3173-5p on PKP3 expression by acting as an miRNA sponge and that PKP3 stabilizes PD-L1 in an OTUB1-dependent manner. The circIGF2BP3/PKP3 axis eventually contributes to the immune escape of NSCLC cells by upregulating PD-L1 expression. We extend the knowledge of circRNA action in antitumor immunity in NSCLC and provide a potential therapeutic approach for improving the efficacy of immunotherapy in NSCLC.

## Supplementary Information

**Additional file 1: Figure S1.** Identification of the circRNA module negatively associated with CD8^+^ TIL infiltration by WGCNA. **A.** Analysis of network topology for soft powers to identify the threshold best fit in the scale-free network. A soft power of 20 was selected to meet the threshold of 0.85. **B.** Heatmap summarizing the proportions of infiltrated immune cell subpopulations in five NSCLC samples in GSE126533 analyzed by CIBERSORT. **C.** Heatmap illustrating the module associations. Red denotes a significant correlation between corresponding modules. **D.** Scatter plot showing the relationship between gene significance and module membership of circRNAs in the turquoise module. circRNAs enclosed in the red box (gene significance > − 0.96, module membership value > 0.96 and q weighted < 0.01) were selected as hub circRNAs. **E.** Relative expression levels of circIGF2BP3 in a panel of NSCLC cell lines, a human bronchial epithelial cell line (16HBE), a human lung epithelial cell line (BEAS-2B) and immune cells (CD45^+^, CD3^+^, CD4^+^ and CD8^+^) isolated from PBMCs. Data represent the mean ± SD.

**Additional file 2: Figure S2.** circIGF2BP3 suppresses antitumor immunity. **A.** Schematic diagram showing the sequences of sh-circIGF2BP3#1 and sh-circIGF2BP3#2. **B-C.** The levels of circIGF2BP3 (left) and linear IGF2BP3 (right) in NSCLC cells transfected with circIGF2BP3-expressing plasmid or circIGF2BP3 shRNA were analyzed by qPCR. **D.** Representative images and statistical quantitation of FACS data of PBMC-mediated elimination of NSCLC cells, as determined by annexin V-FITC and propidium iodide (PI) double labeling. **E.** Schematic diagram showing the construction of the circIGF2BP3-si-Mut plasmid. **F.** Relative viability of the indicated A549 cells after coculturing with PBMCs and viability of cocultured PBMCs, as detected by the CCK-8 assay. **G.** Relative viability of the indicated SK-MES-1 cells after coculturing with PBMCs and viability of cocultured PBMCs detected by CCK-8 assay. **H.** The level of circIGF2BP3 in LLC cells transfected with the circIGF2BP3-expressing plasmid was analyzed by qPCR. Data represent the mean ± SD. *P < 0.05, **P < 0.01, ***P < 0.001. *P* values were determined by unpaired Student’s t test (**B** and **H**) and one-way ANOVA with Tukey’s post hoc test (**B, C, D, F** and **G**).

**Additional file 3: Figure S3.** circIGF2BP3 decreases CCL5 levels in the TME but does not influence MDSCs, TAMs or Treg infiltration. **A.** The mRNA levels of chemokines involved in the recruitment of CD8^+^ T cells in tumors were determined by qPCR. **B-D.** Representative flow cytometry data for CD11b^+^ MDSCs (**B**), CD68^+^CD206^+^ M2-like macrophages (**C**) and CD4^+^Foxp3^+^ Tregs (**D**) in tumors implanted in immunocompetent mice. Data represent the mean ± SD. *P < 0.05, **P < 0.01, ***P < 0.001. *P* values were determined by unpaired Student’s t test.

**Additional file 4: Figure S4.** METTL3 upregulates circIGF2BP3 expression in a YTHDC1-dependent manner. **A-B.** The candidate m^6^A modification sites in circIGF2BP3 predicted by RMBase 2.0 (**A**) and SRAMP (**B**). **C.** Relative abundance of m^6^A-modified circIGF2BP3 in A549 and SW900 cells after treatment with MAO-circIGF2BP3 or MAO-NC, as detected by MeRIP-qPCR. **D.** MeRIP-qPCR analysis of the relative abundance of m^6^A-modified circIGF2BP3 in circIGF2BP3-expressing and circIGF2BP3-m^6^A-Mut-expressing NSCLC cells. **E.** Schematic diagram showing the construction of the circIGF2BP3-m^6^A-Mut plasmid. **F.** The silencing efficiency of si-METTL3 in A549 cells was determined by qRT-PCR (left) and western blotting (right). **G.** The relative expression of METTL14 and circIGF2BP3 in A549 cells transfected with siControl or siMETTL14 was detected by qPCR and western blotting. **H.** The silencing efficiency of si-WTAP in A549 cells was determined by western blotting. **I.** The relative expression of WTAP (left) and circIGF2BP3 (right) in A549 cells transfected with siControl or siMETTL14 was detected by qPCR. **J-K.** Analysis of the relative expression of circIGF2BP3 (**K**) and the relative abundance of m^6^A-modified circIGF2BP3 (**J**) in circIGF2BP3-m^6^A-Mut-expressing NSCLC cells transfected with siControl or siMETTL3. **L.** The relative expression of circIGF2BP3 in SW900 cells transfected with siControl or siYTHDC1 was detected by qPCR. **M.** The silencing efficiency of YTHDC1 in A549 cells was determined by qRT-PCR (left) and western blotting (right). **N.** Time-course qRT-PCR analyses of the relative abundance of circIGF2BP3 in siControl or siYTHDC1 A549 cells treated with actinomycin D (10 μg/ml). **O.** qRT-PCR analysis of circIGF2BP3 abundance in the cytoplasmic and nuclear fractions of A549 cells transfected with siControl or siYTHDC1. Data represent the mean ± SD. *P < 0.05, **P < 0.01, ***P < 0.001. *P* values were determined by unpaired Student’s t test.

**Additional file 5: Figure S5.** The immunosuppressive effect of circIGF2BP3 is mediated through an increase in the expression of its downstream target PKP3. **A.** The relative expression of OVOL1, FAM83H and PAX9 in 68 paired samples of NSCLC in cohort I. GAPDH was used as an internal control. **B.** Correlation between circIGF2BP3 expression and OVOL1, PAX9 or FAM83H expression in 68 paired samples of NSCLC in cohort I. **C.** The relative expression of PKP3 in A549 cells transfected with OEControl or circIGF2BP3-OE, as determined by qRT-PCR. **D.** The silencing efficiency of shPKP3#1 and shPKP3#2 in SW900 cells was determined by qRT-PCR and western blotting. **E.** The levels of PKP3 in H1650 cells transfected with PKP3-overexpressing plasmid were determined by qRT-PCR and western blotting. **F.** The relative abundance of IFN-γ and TNF-α secreted into the medium by activated PBMCs after coculturing with the indicated NSCLC cells was detected by ELISA. **G.** Relative expression levels of PKP3 in a panel of NSCLC cell lines, a human bronchial epithelial cell line (16HBE), a human lung epithelial cell line (BEAS-2B) and immune cells (CD45^+^, CD3^+^, CD4^+^ and CD8^+^) isolated from PBMCs, as determined by qPCR. **H.** Ki-67 (upper) and TUNEL (lower) staining of PBMCs after coculturing with the indicated NSCLC cells. Scale bars, 20 μm. **I.** The mRNA levels of chemokines involved in the recruitment of CD8^+^ T cells in tumors were determined by qPCR. **J.** Representative flow cytometry data for CD11b^+^ MDSCs, CD68^+^CD206^+^ M2-like macrophages and CD4^+^Foxp3^+^ Tregs in tumors implanted in immunocompetent mice. K. Correlation between PKP3 and CCL5 mRNA levels in LUAD (left) and LUSC (right) from the TCGA data set, as analyzed by TIMER 2.0. **L.** The relative expression of PKP3 in LLC cells and infiltrated CD8^+^ T cells was determined by qPCR. *P < 0.05, **P < 0.01, ***P < 0.001. *P* values were determined by unpaired Student’s t test (**A, C, E** and **F**) and one-way ANOVA with Tukey’s post hoc test (**D, F, H, I** and **J**). Correlations were determined by the Pearson correlation test (**B**) and the Spearman correlation test (**K**).

**Additional file 6: Figure S6.** circIGF2BP3 acts as a miRNA sponge for miR-328-3p and miR-3173-5p. **A.** Schematic diagram showing the sequence of wild-type or mutant fragments of the circIGF2BP3 luciferase reporter. **B.** Schematic diagram showing the sequence of wild-type or mutant fragments of the PKP3 luciferase reporter. **C.** The relative expression of PKP3 in A549 cells transfected with miR-328-3p/miR-3173-5p mimics alone or cotransfected with circIGF2BP3. **D.** Schematic diagram showing the construction of the circIGF2BP3-miR-Mut plasmid. Data represent the mean ± SD. *P < 0.05, **P < 0.01, ***P < 0.001. *P* values were determined by one-way ANOVA with Tukey’s post hoc test.

**Additional file 7: Figure S7.** PKP3 increases the protein level of PD-L1. **A.** The relative expression of PD-L1 in A549 cells transfected with shControl or shPKP3 was detected by qRT-PCR. **B.** The relative expression of PD-L1 in PKP3-overexpressing SK-MES-1 cells was detected by qRT-PCR. **C.** Correlation analysis between PKP3 and PD-L1 mRNA levels in NSCLC samples from the TCGA data set. **D-I.** Western blot analysis of PD-L1 levels in A549 cells transfected with the indicated constructs. **J.** Western blot analysis of PD-L1 levels in CTRL and sgCD274 LLC cells. Data represent the mean ± SD. *P* values were determined by unpaired Student’s t test (**A** and **B**). Correlations were determined by the Pearson correlation test (**B**).

**Additional file 8: Figure S8.** PKP3/PD-L1 axis inhibition enhances the efficacy of anti-PD-1 therapy. **A.** Schematic diagram showing the grouping and treatment plan of the in vivo study: PD-L1 WT or PD-L1 KO mice were inoculated with sh-mouse PKP3 LLC cells, and the tumor volume was measured at the indicated time points. **B.** Left panel: FACS histogram showing PD-L1 levels on myeloid cells (CD11b^+^) from PD-L1 KO or WT mice. Right panel: FACS histogram showing PD-L1 levels in LLC cells with or without mouse PKP3 silencing. **C.** Time-course evaluation of tumor volumes of each group measured every 3 days. **D.** Quantification of the flow cytometry data demonstrating the percentage of CD3^+^ cells among CD45^+^ T cells; CD8^+^ T cells among CD3^+^ cells; and GZMB^+^ T cells among CD8^+^ cells. **E.** Schematic diagram showing the grouping and treatment plan of the in vivo study: C57BL/6 mice were inoculated with 10^6^ CTRL, PKP3-OE, or circIGF2BP3-OE LLC cells with or without anti-PD-1 treatment, and the tumor volume was measured at the indicated time points. **F.** Left panel: PD-L1 expression in implanted LLC cells of each group was analyzed by FACS. Right panel: PD-L1 expression in CD45^+^ cells isolated from tumor tissues of each group was analyzed by FACS. **G.** Time-course evaluation of tumor volumes of each group measured every 3 days. **H.** Quantification of the flow cytometry data demonstrating the percentage of CD3^+^ cells among CD45^+^ T cells; CD8^+^ T cells among CD3^+^ cells; and GZMB^+^ T cells among CD8^+^ cells. Data represent the mean ± SD. *P* values were by one-way ANOVA with Tukey’s post hoc test.

**Additional file 9: Table S1.** Antibodies and peptides used in this study. **Table S2**. Reagents used in this study. **Table S3**. Oligonucleotides used in this study. **Table S8.** Correlation between circIGF2BP3 levels and different clinical characteristics of NSCLC in cohort I.

**Additional file 10: Table S4**

**Additional file 11: Table S5**

**Additional file 12: Table S6**

**Additional file 13: Table S7**

## Data Availability

The data supporting the conclusions of this article have been provided in this article and its additional files. In addition, all data from this study can be obtained from the corresponding author upon reasonable request.

## Abbreviations

circRNAs: Circular RNAs
NSCLC: Non-small-cell lung carcinoma
RIP: RNA immunoprecipitation
FISH: Fluorescence in situ hybridization
PKP3: Plakophilin 3
IP: Immunoprecipitation
LUAD: Lung adenocarcinoma
LUSC: Lung squamous cell carcinoma
ICBs: Immune checkpoint blockade therapies
PD-1: Programmed cell death protein 1
PD-L1: programmed cell death-ligand 1
ncRNAs: Noncoding RNAs
METTL3: Methyltransferase like 3
FBS: Fetal bovine serum
shRNA: Short hairpin RNAs
UTRs: Untranslated regions
IF: Immunofluorescence
PBMCs: Peripheral blood monocyte cells
BSA: Bovine serum albumin
PI: Propidium iodide
FACS: Fluorescence-activated cell sorting
SDS: Sodium dodecyl sulfate
MeRIP: Methylated RNA immunoprecipitation
IHC: Immunohistochemistry
HRP: Horseradish peroxidase
CHX: Cycloheximide
LLC: Lewis lung carcinoma
TILs: Tumor-infiltrating lymphocytes
GEO: Gene Expression Omnibus
WGCNA: Weighted gene coexpression network analysis
TCGA: The Cancer Genome Atlas
ceRNA: Competing endogenous RNA
GzmB: Granzyme B
MAOs: Morpholino antisense oligos
WT: Wild-type
YTHDC1: YTH domain containing 1
OS: Overall survival
OTUB1: Otubain-1
FXR1: Fragile X mental retardation syndrome-related protein 1
TAM: Tumor-associated macrophage
TME: Tumor microenvironment
m^6^A: N^6^-methyladenosine
KO: Knockout
MDSC: myeloid-derived suppressor cell
K-M: Kaplan-Meier
