# Supplementary figures and images for: N6-methyladenosine-modified circIGF2BP3 inhibits CD8+ T-cell responses to facilitate tumor immune evasion by promoting the deubiquitination of PD-L1 in non-small cell lung cancer
Source: Mol Cancer. 2021 Aug 20;20:105. doi: 10.1186/s12943-021-01398-4 (PMC8377850; doi:10.1186/s12943-021-01398-4)

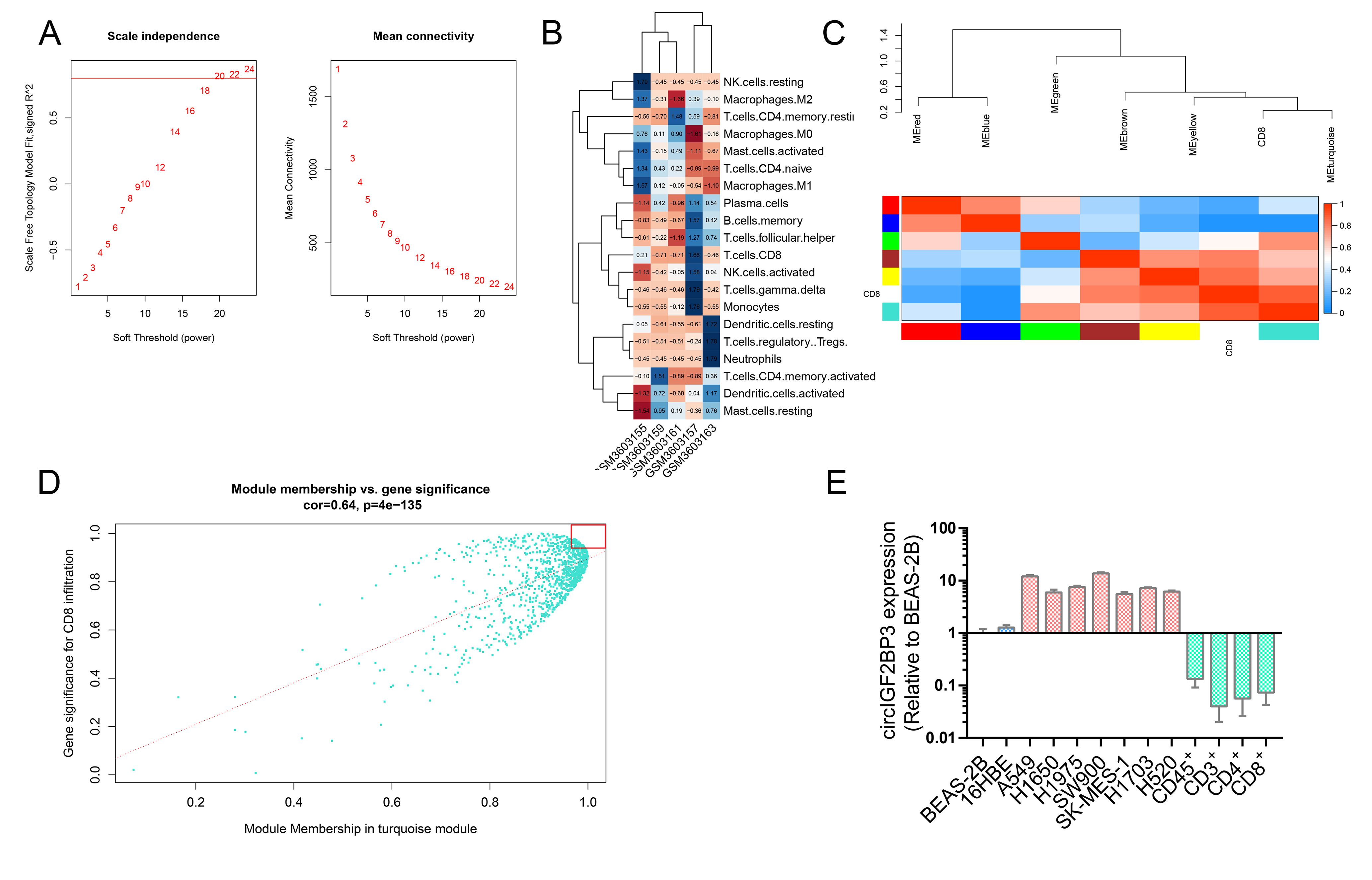

Supplement: Supplementary file 1 — Additional file 1: Figure S1. Identification of the circRNA module negatively associated with CD8+ TIL infiltration by WGCNA. A. Analysis of network topology for soft powers to identify the threshold best fit in the scale-free network. A soft power of 20 was selected to meet the threshold of 0.85. B. Heatmap summarizing the proportions of infiltrated immune cell subpopulations in five NSCLC samples in GSE126533 analyzed by CIBERSORT. C. Heatmap illustrating the module associations. Red denotes a significant correlation between corresponding modules. D. Scatter plot showing the relationship between gene significance and module membership of circRNAs in the turquoise module. circRNAs enclosed in the red box (gene significance > − 0.96, module membership value > 0.96 and q weighted < 0.01) were selected as hub circRNAs. E. Relative expression levels of circIGF2BP3 in a panel of NSCLC cell lines, a human bronchial epithelial cell line (16HBE), a human lung epithelial cell line (BEAS-2B) and immune cells (CD45+, CD3+, CD4+ and CD8+) isolated from PBMCs. Data represent the mean ± SD. [file 12943_2021_1398_MOESM1_ESM.tif]

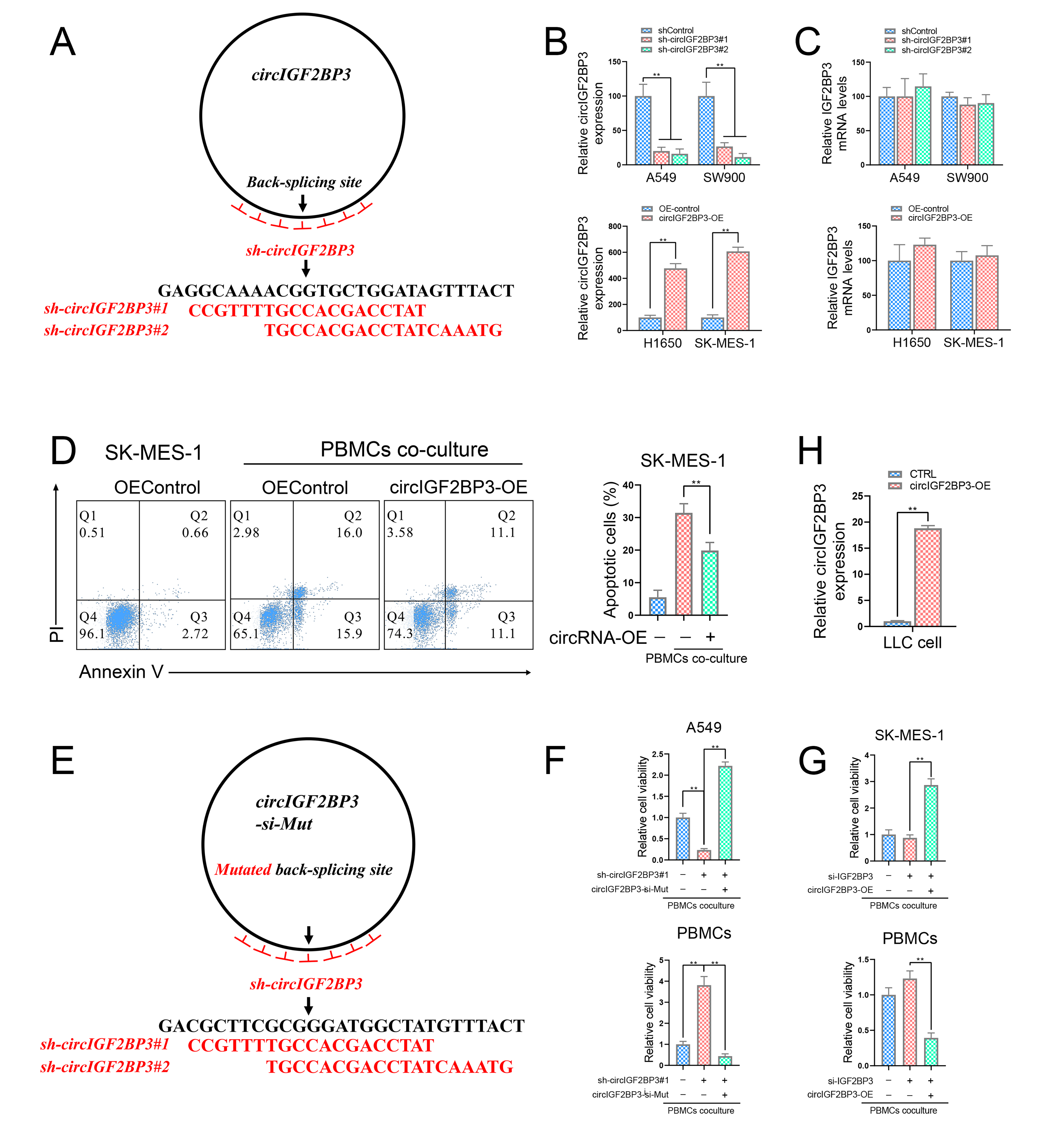

Supplement: Supplementary file 2 — Additional file 2: Figure S2. circIGF2BP3 suppresses antitumor immunity. A. Schematic diagram showing the sequences of sh-circIGF2BP3#1 and sh-circIGF2BP3#2. B-C. The levels of circIGF2BP3 (left) and linear IGF2BP3 (right) in NSCLC cells transfected with circIGF2BP3-expressing plasmid or circIGF2BP3 shRNA were analyzed by qPCR. D. Representative images and statistical quantitation of FACS data of PBMC-mediated elimination of NSCLC cells, as determined by annexin V-FITC and propidium iodide (PI) double labeling. E. Schematic diagram showing the construction of the circIGF2BP3-si-Mut plasmid. F. Relative viability of the indicated A549 cells after coculturing with PBMCs and viability of cocultured PBMCs, as detected by the CCK-8 assay. G. Relative viability of the indicated SK-MES-1 cells after coculturing with PBMCs and viability of cocultured PBMCs detected by CCK-8 assay. H. The level of circIGF2BP3 in LLC cells transfected with the circIGF2BP3-expressing plasmid was analyzed by qPCR. Data represent the mean ± SD. *P < 0.05, **P < 0.01, ***P < 0.001. P values were determined by unpaired Student’s t test (B and H) and one-way ANOVA with Tukey’s post hoc test (B, C, D, F and G). [file 12943_2021_1398_MOESM2_ESM.tif]

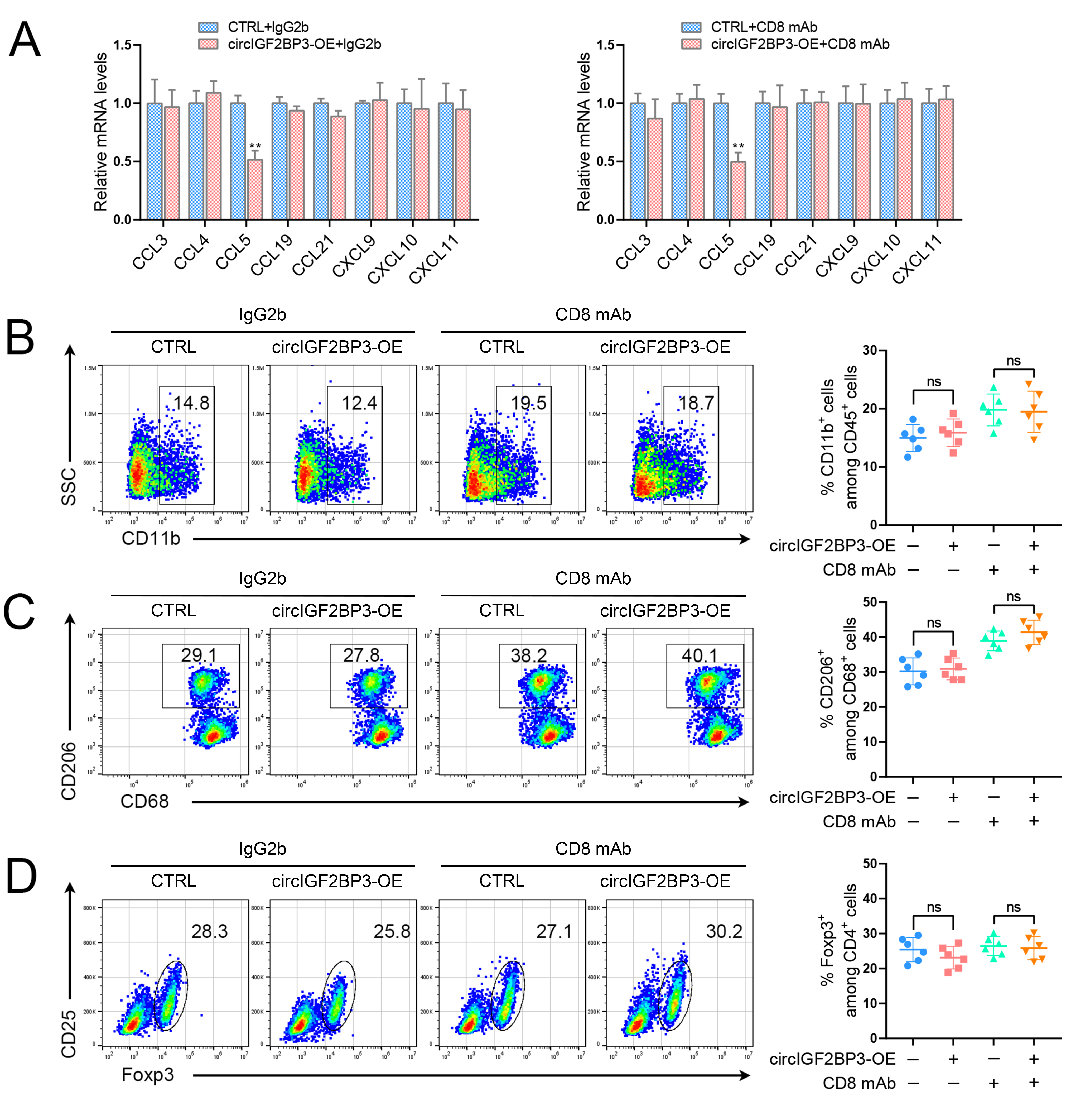

Supplement: Supplementary file 3 — Additional file 3: Figure S3. circIGF2BP3 decreases CCL5 levels in the TME but does not influence MDSCs, TAMs or Treg infiltration. A. The mRNA levels of chemokines involved in the recruitment of CD8+ T cells in tumors were determined by qPCR. B-D. Representative flow cytometry data for CD11b+ MDSCs (B), CD68+CD206+ M2-like macrophages (C) and CD4+Foxp3+ Tregs (D) in tumors implanted in immunocompetent mice. Data represent the mean ± SD. *P < 0.05, **P < 0.01, ***P < 0.001. P values were determined by unpaired Student’s t test. [file 12943_2021_1398_MOESM3_ESM.tif]

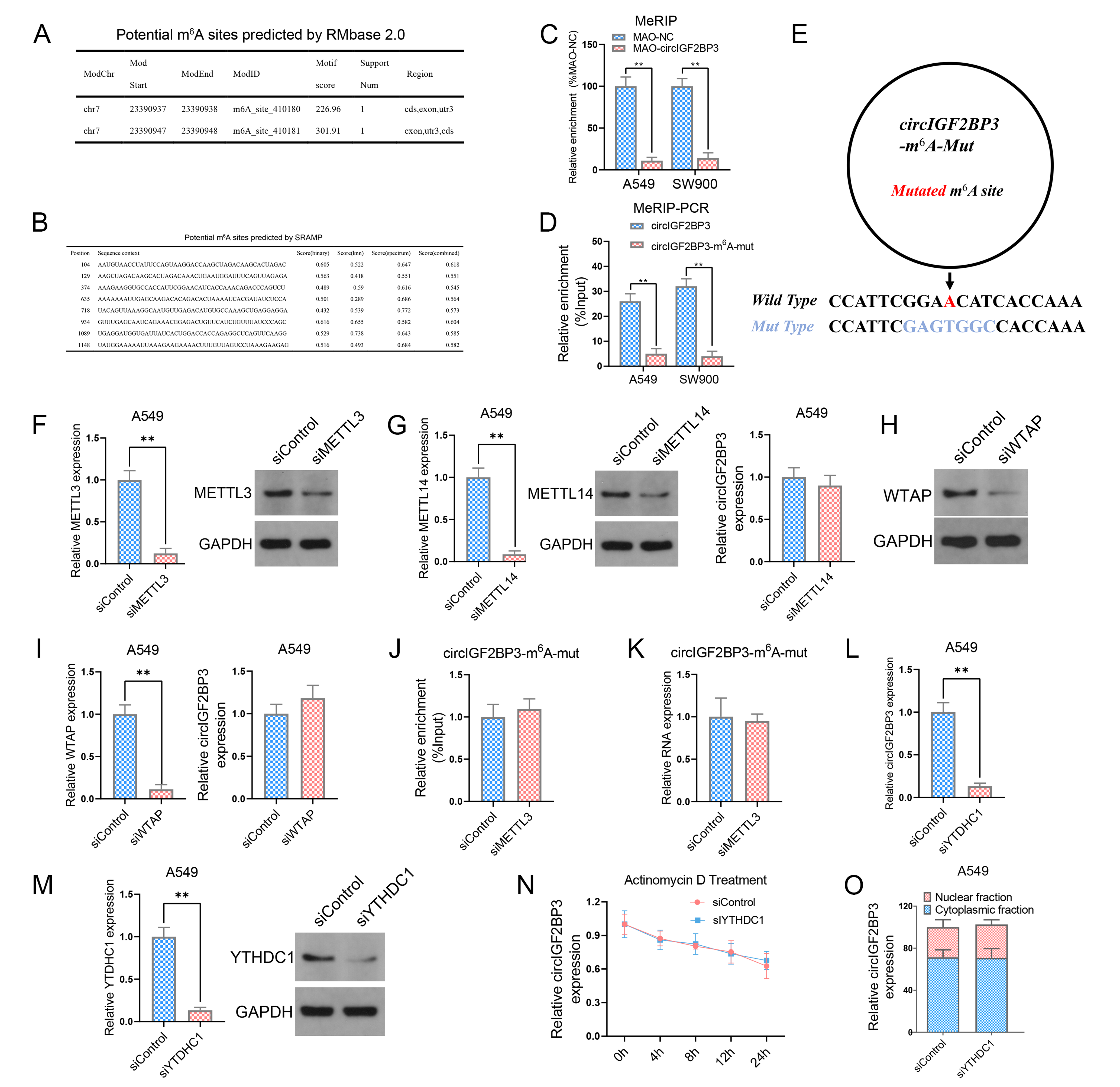

Supplement: Supplementary file 4 — Additional file 4: Figure S4. METTL3 upregulates circIGF2BP3 expression in a YTHDC1-dependent manner. A-B. The candidate m6A modification sites in circIGF2BP3 predicted by RMBase 2.0 (A) and SRAMP (B). C. Relative abundance of m6A-modified circIGF2BP3 in A549 and SW900 cells after treatment with MAO-circIGF2BP3 or MAO-NC, as detected by MeRIP-qPCR. D. MeRIP-qPCR analysis of the relative abundance of m6A-modified circIGF2BP3 in circIGF2BP3-expressing and circIGF2BP3-m6A-Mut-expressing NSCLC cells. E. Schematic diagram showing the construction of the circIGF2BP3-m6A-Mut plasmid. F. The silencing efficiency of si-METTL3 in A549 cells was determined by qRT-PCR (left) and western blotting (right). G. The relative expression of METTL14 and circIGF2BP3 in A549 cells transfected with siControl or siMETTL14 was detected by qPCR and western blotting. H. The silencing efficiency of si-WTAP in A549 cells was determined by western blotting. I. The relative expression of WTAP (left) and circIGF2BP3 (right) in A549 cells transfected with siControl or siMETTL14 was detected by qPCR. J-K. Analysis of the relative expression of circIGF2BP3 (K) and the relative abundance of m6A-modified circIGF2BP3 (J) in circIGF2BP3-m6A-Mut-expressing NSCLC cells transfected with siControl or siMETTL3. L. The relative expression of circIGF2BP3 in SW900 cells transfected with siControl or siYTHDC1 was detected by qPCR. M. The silencing efficiency of YTHDC1 in A549 cells was determined by qRT-PCR (left) and western blotting (right). N. Time-course qRT-PCR analyses of the relative abundance of circIGF2BP3 in siControl or siYTHDC1 A549 cells treated with actinomycin D (10 μg/ml). O. qRT-PCR analysis of circIGF2BP3 abundance in the cytoplasmic and nuclear fractions of A549 cells transfected with siControl or siYTHDC1. Data represent the mean ± SD. *P < 0.05, **P < 0.01, ***P < 0.001. P values were determined by unpaired Student’s t test. [file 12943_2021_1398_MOESM4_ESM.tif]

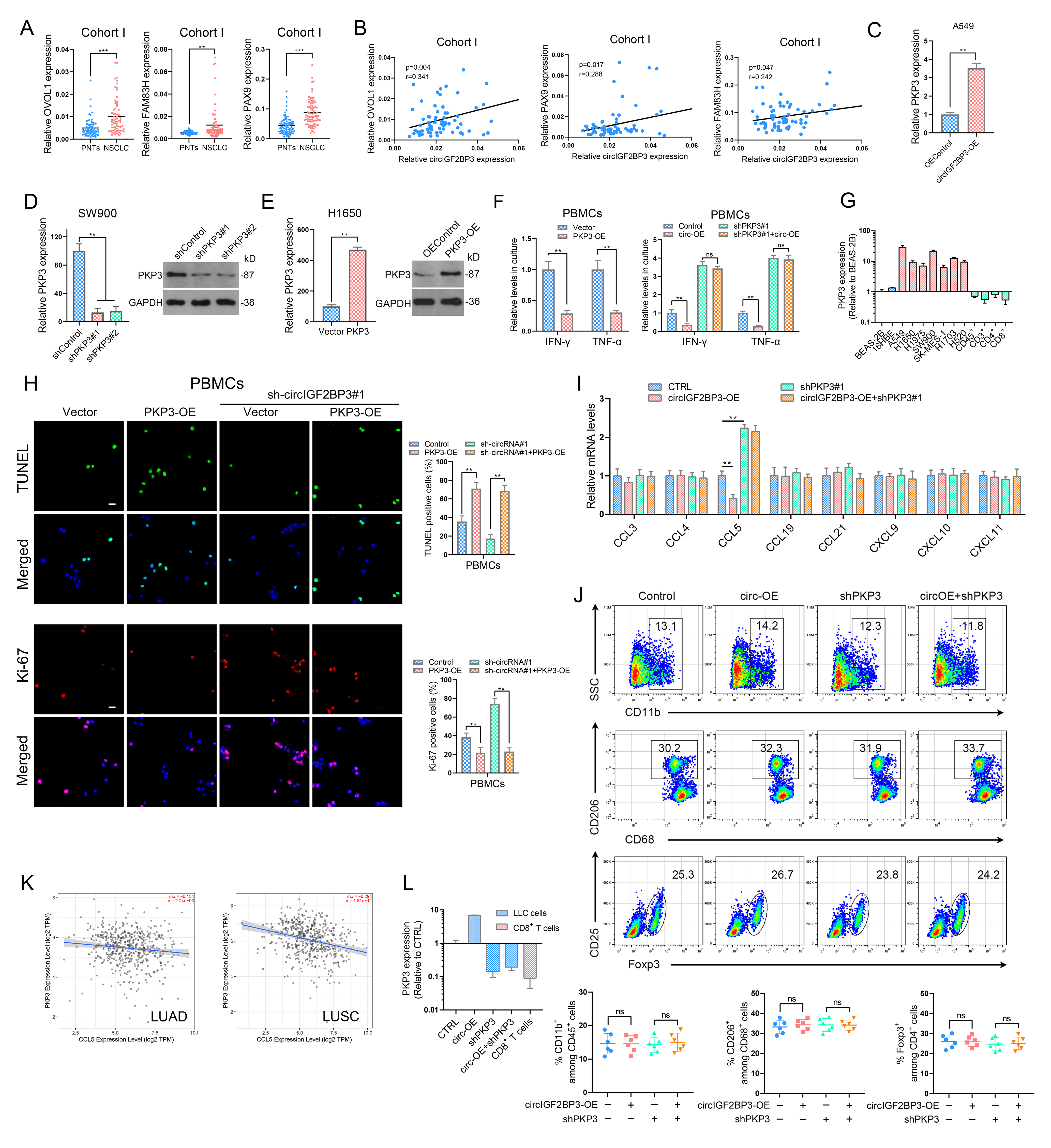

Supplement: Supplementary file 5 — Additional file 5: Figure S5. The immunosuppressive effect of circIGF2BP3 is mediated through an increase in the expression of its downstream target PKP3. A. The relative expression of OVOL1, FAM83H and PAX9 in 68 paired samples of NSCLC in cohort I. GAPDH was used as an internal control. B. Correlation between circIGF2BP3 expression and OVOL1, PAX9 or FAM83H expression in 68 paired samples of NSCLC in cohort I. C. The relative expression of PKP3 in A549 cells transfected with OEControl or circIGF2BP3-OE, as determined by qRT-PCR. D. The silencing efficiency of shPKP3#1 and shPKP3#2 in SW900 cells was determined by qRT-PCR and western blotting. E. The levels of PKP3 in H1650 cells transfected with PKP3-overexpressing plasmid were determined by qRT-PCR and western blotting. F. The relative abundance of IFN-γ and TNF-α secreted into the medium by activated PBMCs after coculturing with the indicated NSCLC cells was detected by ELISA. G. Relative expression levels of PKP3 in a panel of NSCLC cell lines, a human bronchial epithelial cell line (16HBE), a human lung epithelial cell line (BEAS-2B) and immune cells (CD45+, CD3+, CD4+ and CD8+) isolated from PBMCs, as determined by qPCR. H. Ki-67 (upper) and TUNEL (lower) staining of PBMCs after coculturing with the indicated NSCLC cells. Scale bars, 20 μm. I. The mRNA levels of chemokines involved in the recruitment of CD8+ T cells in tumors were determined by qPCR. J. Representative flow cytometry data for CD11b+ MDSCs, CD68+CD206+ M2-like macrophages and CD4+Foxp3+ Tregs in tumors implanted in immunocompetent mice. K. Correlation between PKP3 and CCL5 mRNA levels in LUAD (left) and LUSC (right) from the TCGA data set, as analyzed by TIMER 2.0. L. The relative expression of PKP3 in LLC cells and infiltrated CD8+ T cells was determined by qPCR. *P < 0.05, **P < 0.01, ***P < 0.001. P values were determined by unpaired Student’s t test (A, C, E and F) and one-way ANOVA with Tukey’s post hoc test (D, F, H, I and J). Correlation [file 12943_2021_1398_MOESM5_ESM.tif]

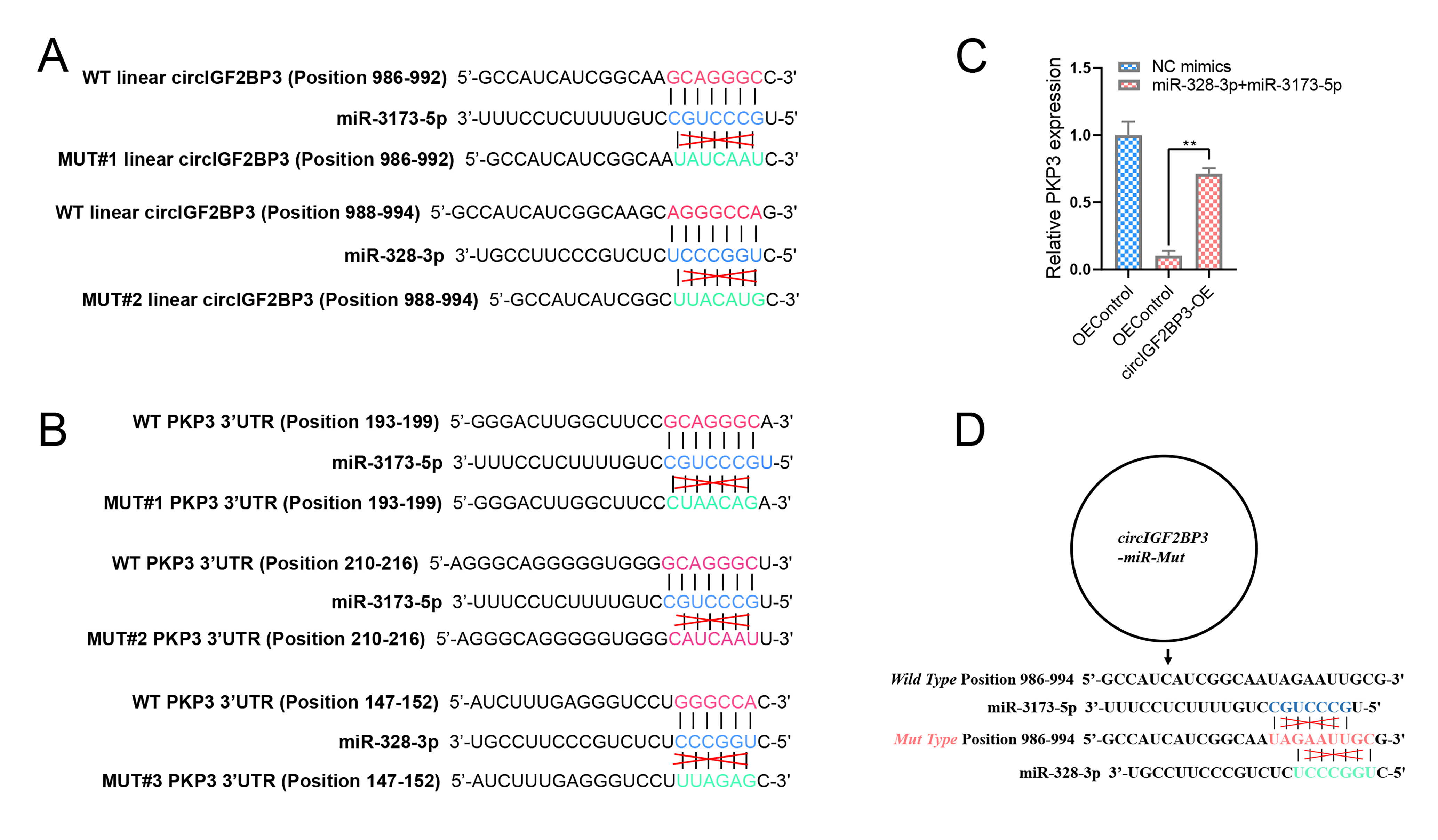

Supplement: Supplementary file 6 — Additional file 6: Figure S6. circIGF2BP3 acts as a miRNA sponge for miR-328-3p and miR-3173-5p. A. Schematic diagram showing the sequence of wild-type or mutant fragments of the circIGF2BP3 luciferase reporter. B. Schematic diagram showing the sequence of wild-type or mutant fragments of the PKP3 luciferase reporter. C. The relative expression of PKP3 in A549 cells transfected with miR-328-3p/miR-3173-5p mimics alone or cotransfected with circIGF2BP3. D. Schematic diagram showing the construction of the circIGF2BP3-miR-Mut plasmid. Data represent the mean ± SD. *P < 0.05, **P < 0.01, ***P < 0.001. P values were determined by one-way ANOVA with Tukey’s post hoc test. [file 12943_2021_1398_MOESM6_ESM.tif]

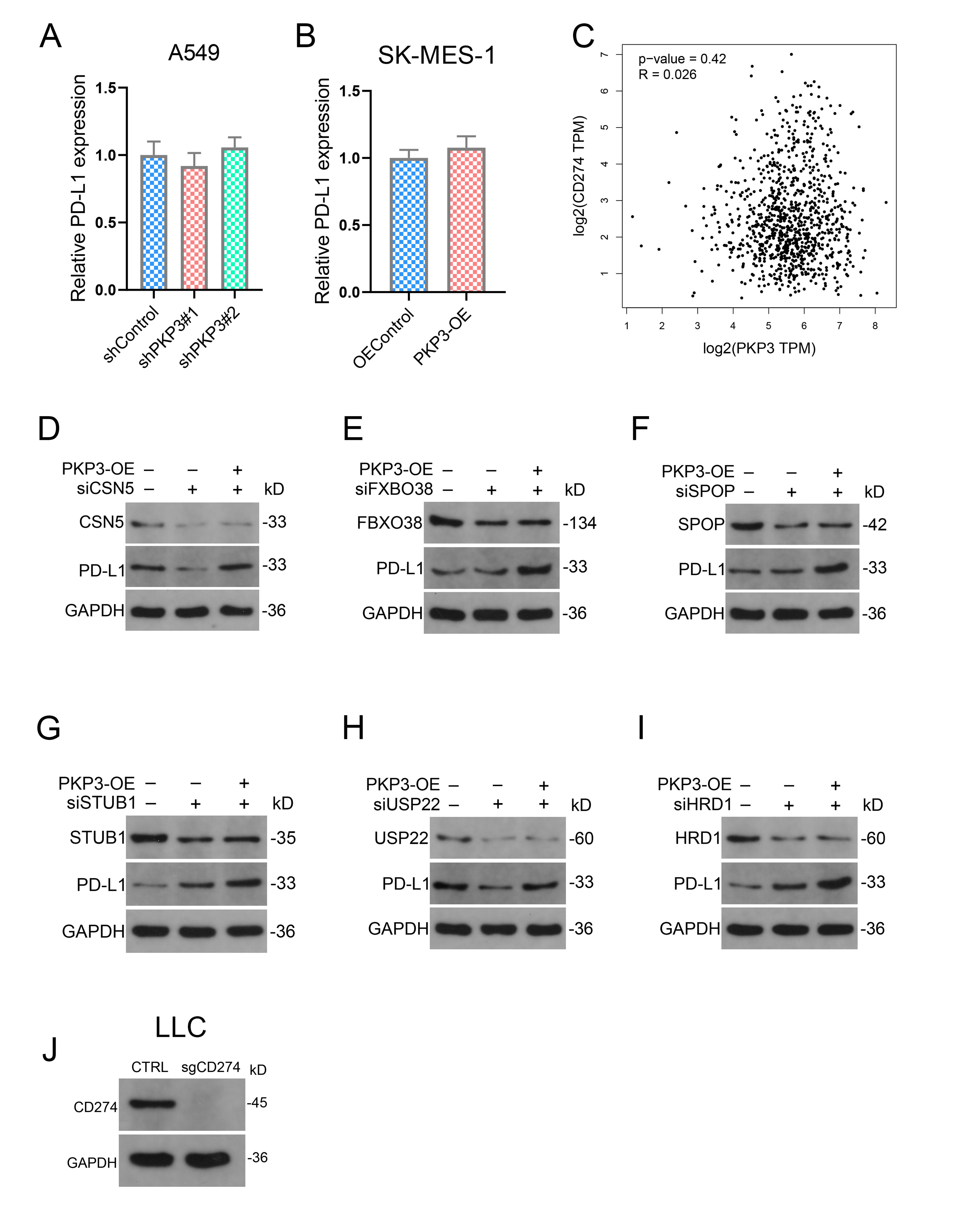

Supplement: Supplementary file 7 — Additional file 7: Figure S7. PKP3 increases the protein level of PD-L1. A. The relative expression of PD-L1 in A549 cells transfected with shControl or shPKP3 was detected by qRT-PCR. B. The relative expression of PD-L1 in PKP3-overexpressing SK-MES-1 cells was detected by qRT-PCR. C. Correlation analysis between PKP3 and PD-L1 mRNA levels in NSCLC samples from the TCGA data set. D-I. Western blot analysis of PD-L1 levels in A549 cells transfected with the indicated constructs. J. Western blot analysis of PD-L1 levels in CTRL and sgCD274 LLC cells. Data represent the mean ± SD. P values were determined by unpaired Student’s t test (A and B). Correlations were determined by the Pearson correlation test (B). [file 12943_2021_1398_MOESM7_ESM.tif]

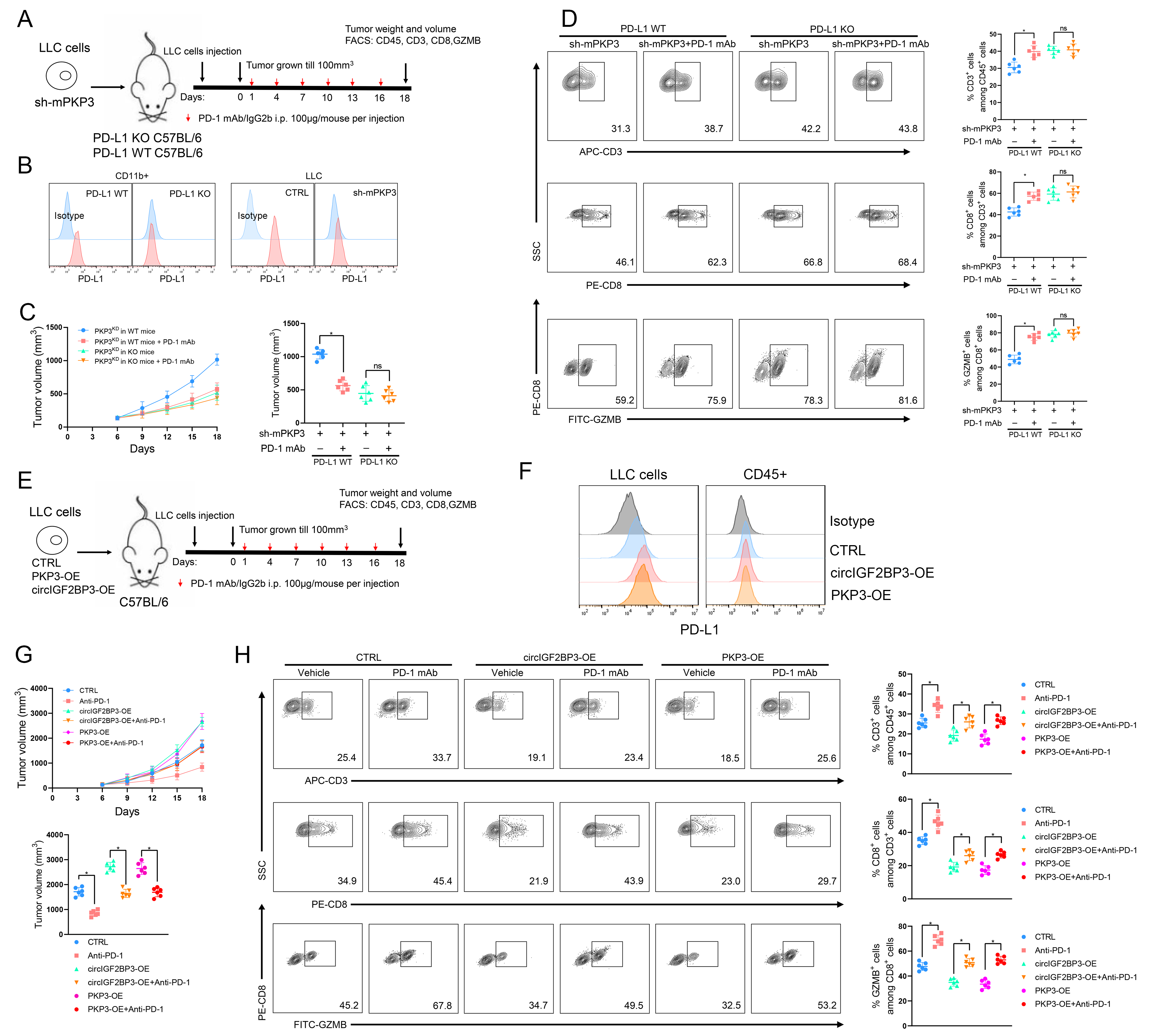

Supplement: Supplementary file 9 — Additional file 9: Table S1. Antibodies and peptides used in this study. Table S2. Reagents used in this study. Table S3. Oligonucleotides used in this study. Table S8. Correlation between circIGF2BP3 levels and different clinical characteristics of NSCLC in cohort I. [file 12943_2021_1398_MOESM9_ESM.tif]
