# Supplementary material for: N6-methyladenosine-modified circIGF2BP3 inhibits CD8+ T-cell responses to facilitate tumor immune evasion by promoting the deubiquitination of PD-L1 in non-small cell lung cancer
Source: Mol Cancer. 2021 Aug 20;20:105. doi: 10.1186/s12943-021-01398-4 (PMC8377850; doi:10.1186/s12943-021-01398-4)
Supplement: Supplementary file 8 — Additional file 8: Figure S8. PKP3/PD-L1 axis inhibition enhances the efficacy of anti-PD-1 therapy. A. Schematic diagram showing the grouping and treatment plan of the in vivo study: PD-L1 WT or PD-L1 KO mice were inoculated with sh-mouse PKP3 LLC cells, and the tumor volume was measured at the indicated time points. B. Left panel: FACS histogram showing PD-L1 levels on myeloid cells (CD11b+) from PD-L1 KO or WT mice. Right panel: FACS histogram showing PD-L1 levels in LLC cells with or without mouse PKP3 silencing. C. Time-course evaluation of tumor volumes of each group measured every 3 days. D. Quantification of the flow cytometry data demonstrating the percentage of CD3+ cells among CD45+ T cells; CD8+ T cells among CD3+ cells; and GZMB+ T cells among CD8+ cells. E. Schematic diagram showing the grouping and treatment plan of the in vivo study: C57BL/6 mice were inoculated with 106 CTRL, PKP3-OE, or circIGF2BP3-OE LLC cells with or without anti-PD-1 treatment, and the tumor volume was measured at the indicated time points. F. Left panel: PD-L1 expression in implanted LLC cells of each group was analyzed by FACS. Right panel: PD-L1 expression in CD45+ cells isolated from tumor tissues of each group was analyzed by FACS. G. Time-course evaluation of tumor volumes of each group measured every 3 days. H. Quantification of the flow cytometry data demonstrating the percentage of CD3+ cells among CD45+ T cells; CD8+ T cells among CD3+ cells; and GZMB+ T cells among CD8+ cells. Data represent the mean ± SD. P values were by one-way ANOVA with Tukey’s post hoc test. [file 12943_2021_1398_MOESM8_ESM.docx]

**Supplementary table**

**Table S1. Antibodies and peptides used in this study.**

| **Antibodies or peptides** | **Application** | **Source** | **Identifier** |
| --- | --- | --- | --- |
| Human Anti-PD-L1 | WB | Abcam | Cat# ab205921 |
| Human Anti-CD8 | IHC | Abcam | Cat# ab217344 |
| Human Anti-PKP3 | IF | Abcam | Cat# ab151410 |
| Human Anti-PKP3 | WB, IF, IHC | Abcam | Cat# ab109441 |
| Human Anti-Perforin | WB | Abcam | Cat# ab75573 |
| Human Anti-GzmB | WB | Abcam | Cat# ab255598 |
| Human Anti-Ki67 | IF | Abcam | Cat# ab15580 |
| In vivo mAb anti-mouse-CD8a | in vivo study | BioXcel | Cat# BP0117 |
| IgG2b isotype | in vivo study | BioXcel | Cat# BP0090 |
| Mouse Anti-GzmB | FACS | eBioscience | Cat# NGZB |
| Mouse Anti-perforin | FACS | eBioscience | Cat# eBioOMAKD |
| Mouse Anti-IFN-γ | FACS | Miltenyi Biotec | Cat# REA638 |
| Mouse Anti-TNF-α | FACS | Miltenyi Biotec | Cat# MP6XT22 |
| Mouse Anti-CD8α | IF, FACS | Abcam | Cat# ab217344 |
| Mouse Anti-CD3/APC | FACS | Abcam | Cat# ab210190 |
| Mouse Anti-CD8/PE | FACS | Abcam | Cat# ab272343 |
| Mouse Anti-CD45/PerCP-Cy5.5 | FACS | Abcam | Cat# ab210187 |
| Mouse Anti-CD11b/PE | FACS | Abcam | Cat# ab133357 |
| Mouse Anti-CD4/FITC | FACS | Abcam | Cat# ab269349 |
| Mouse Anti-Foxp3/PE-Cy7 | FACS | Abcam | Cat# ab210232 |
| Mouse Anti-CD25/PE | FACS | Abcam | Cat# ab24933 |
| Mouse Anti-CD68/PE | FACS | Abcam | Cat# ab216701 |
| Mouse Anti-CD206/Alexa Fluor 700 | FACS | eBioscience | Cat# 56-2061-82 |
| Mouse Anti-PKP3 | IF | Abcam | Cat# ab247865 |
| Mouse Anti-PD-L1 | IF, FACS | Abcam | Cat# ab213480 |
| Human Anti-m6A | MeRIP | Abcam | Cat# ab208577 |
| Human Anti-METTL3 | WB, RIP | Abcam | Cat# ab195352 |
| Human Anti-METTL14 | WB | Abcam | Cat# ab220030 |
| Human Anti-WTAP | WB | Abcam | Cat# ab195380 |
| Human Anti-YTHDC1 | WB, RIP | Abcam | Cat# ab220159 |
| Human Anti-AGO2 | RIP | Abcam | Cat# ab186733 |
| Human Anti-IgG/Alexa Fluor 647 | IF | Abcam | Cat# ab200623 |
| Mouse Anti-IgG/Alexa Fluor 647 | IF | Abcam | Cat# ab150115 |
| Mouse Anti-IgG/Alexa Fluor 488 | IF | Abcam | Cat# ab150113 |
| Rabbit Anti-IgG/Alexa Fluor 647 | IF | Abcam | Cat# ab150063 |
| Rabbit Anti-IgG/Alexa Fluor 488 | IF | Abcam | Cat# ab150081 |
| Recombinant human PD-1 Fc protein | IF | Abcam | Cat# ab216233 |
| Human Anti-OTUB1 | WB, IP, IF | Abcam | Cat# ab175200 |
| Human Anti-CSN5 | WB | Abcam | Cat# ab12323 |
| Human Anti-STUB1 | WB | Abcam | Cat# ab109103 |
| Human Anti-USP22 | WB | Abcam | Cat# ab195289 |
| Human Anti-SPOP | WB | Abcam | Cat# ab137537 |
| Human Anti-FBXO38 | WB | Abcam | Cat# ab87729 |
| Human Anti-HRD1 | WB | Abcam | Cat# ab170901 |
| Human Anti-GAPDH | WB | Abcam | Cat# ab8245 |
| Human Anti-Ubiquitin | Ubiqutination assay | Abcam | Cat# ab134953 |
| Anti-Myc tag | Ubiqutination assay | Abcam | Cat# ab32 |
| Anti-DDDDK tag | Ubiqutination assay | Abcam | Cat# ab205606 |
| Anti-V5 tag | Ubiqutination assay | Abcam | Cat# ab27671 |
| Human Anti-FXR1 | WB, IP | Abcam | Cat# ab129089 |
| In vivo mAb anti-mouse PD-1 | in vivo | BioXcell | Cat# BE0146 |

**Table S2. Reagents used in this study.**

| **Reagent** | **Application** | **Source** | **Identifier** |
| --- | --- | --- | --- |
| RNase R | qRT-PCR | Geneseed | Cat# R0301 |
| Actinomycin | qRT-PCR | MedChemExpress | Cat# HY-12574 |
| MG132 | WB | MedChemExpress | Cat# HY-13259 |
| CHX | WB | Sigma | Cat# C7698 |
| TRIzol | RNA extraction | Invitrogen | Cat# 15596018 |
| Lipofectamine 3000 | Transfection | Thermo Scientific | Cat# L3000015 |

**Table S3. Oligonucleotides used in this study.**

| **Name** | **Sequence** | **Application** |
| --- | --- | --- |
| sh-circIGF2BP3#1 | CCGTTTTGCCACGACCTAT | RNA interfering |
| sh-circIGF2BP3#2 | TGCCACGACCTATCAAATG | RNA interfering |
| shPKP3#1 | CCGAAAGCTCATCTTCATCAA | RNA interfering |
| shPKP3#2 | CGCTAGGAACAAGGACGAGAT | RNA interfering |
| siIGF2BP3 | TCTGCACTTGACAAATTCTGA | RNA interfering |
| siMETTL3 | GCCTTAACATTGCCCACTGAT | RNA interfering |
| siMETTL14 | CCCTAAACTTAGGGAACTCAT | RNA interfering |
| siWTAP | ATGGCAAGAGATGAGTTAATT | RNA interfering |
| siYTHDC1 | TGGATTTGCAGGCGTGAATTA | RNA interfering |
| siOTUB1 | AGGAGTATGCTGAAGATGACA | RNA interfering |
| siCSN5 | CCAGACTATTCCACTTAATAA | RNA interfering |
| siSTUB1 | GAAGAGGAAGAAGCGAGACAT | RNA interfering |
| siUSP22 | AGCTACCAGGAGTCCACAAAG | RNA interfering |
| siSPOP | CACAGATCAAGGTAGTGAAAT | RNA interfering |
| siFBXO38 | GAATGTCTTTCCCGGAAGCTA | RNA interfering |
| siHRD1 | TGAATGCTTAATCCCGGGAAA | RNA interfering |
| siFXR1 | GTATCATATTGCCTATCTAAA | RNA interfering |
| MAO-NC | CCTACCGGGGGACGCCATGTGAGG | MAOs |
| MAO-circIGF2BP3 | ACCATTCGGAACATCACCAAACAG | MAOs |
| shPKP3-Mouse | CCAGAGGTCAATATCACACTA | RNA interfering |
| circIGF2BP3 primers (Divergent) | F: GTCAGTGTTCACTTGCTCACAGCT | qRT-PCR |
|  | R: AGGCTCAGTTCAAGGCTCAGGGA | qRT-PCR |
| circIGF2BP3 primers (Convergent) | F: GAGATTATGCATAAGGAAGCTCAAG | qRT-PCR |
|  | R: GCTAAAATCTTCAAGGGGATCTC | qRT-PCR |
| hsa_circ_0026427 primers | F: GGTCGTCCCCATGTCTGCCTGCTG | qRT-PCR |
|  | R: CTGGAAGGGCTGGAGGATGCCCTGC | qRT-PCR |
| hsa_circ_0026416 primers | F: GTCTGAGATGTGGGTCTGCATCTGG | qRT-PCR |
|  | R: CAGGAGCTGATGAATGTCAAGCTGG | qRT-PCR |
| hsa_circ_0026481 primers | F: CACCTTGTAGGACTTCTGGGTCACC | qRT-PCR |
|  | R: AAGACCCTCAACAACAAGTTTGC | qRT-PCR |
| IGF2BP3 primers | F: ACTGCACGGGAAACCCATAG | qRT-PCR |
|  | R: ACTATCCAGCACCTCCCACT | qRT-PCR |
| KRT6C primers | F: CTATACCTGCCCCATCTGAGC | qRT-PCR |
|  | R: TAAGCCCCAGGCGATTTTCA | qRT-PCR |
| KRT8 primers | F: AGCTTCTCCGCTCCTTCTAGG | qRT-PCR |
|  | R: CAGGCTCTGGTTGACCGTAA | qRT-PCR |
| Perforin primers | F: CTATACGGGATTCCAGCTCCA | qRT-PCR |
|  | R: CATTGCTGGTGGGCTTAGGA | qRT-PCR |
| Granzyme B primers | F: GATCATCGGGGGACATGAGG | qRT-PCR |
|  | R: GGTCGGCTCCTGTTCTTTGA | qRT-PCR |
| pre IGF2BP3 mRNA primers | F: TGCTTCTATGAATGTTAGTTTTG | qRT-PCR |
|  | R: TGTGCTTGAAGCTGCAACAGTA | qRT-PCR |
| GAPDH primers | F: CCATGGGGAAGGTGAAGGTC | qRT-PCR |
|  | R: AGTGATGGCATGGACTGTGG | qRT-PCR |
| U1 primers | F: GGGAGATACCATGATCACGAAGGT | qRT-PCR |
|  | R: CCACAAATTATGCAGTCGAGTTTCCC | qRT-PCR |
| PAX9 primers | F: TTTCATCGGGGCACAGACTT | qRT-PCR |
|  | R: CACGGAGGGCACATTGTACT | qRT-PCR |
| OVOL1 primers | F: CTCGTTCTCAGGGAAGACGG | qRT-PCR |
|  | R: GTGTGAGTTCGGACGTGTCT | qRT-PCR |
| FAM83H primers | F: CCAGAAGATGCCCCTTGGAG | qRT-PCR |
|  | R: TGCTGGATACCAGGAGGACA | qRT-PCR |
| PKP3 primers | F: GGACGGTAACTTCCTGCTGT | qRT-PCR |
|  | R: TCAAAACCGCTGCTGAGTCT | qRT-PCR |
| PD-L1 primers | F: TTGCTGAACGCCCCATACAA | qRT-PCR |
|  | R: TGTCCCGTTCCAACACTGAG | qRT-PCR |
| OTUB1 primers | F: AGAGCACCTCCGACTACCTT | qRT-PCR |
|  | R: CTCCTTGACAGTCCGTCCAC | qRT-PCR |
| FXR1 primers | F: TGCCTCACAAAGTGAGTTGG | qRT-PCR |
|  | R: CCCACATGGCTCTTGGTCAT | qRT-PCR |
| CCL3 primers | F: ATTCCGTCACCTGCTCAGAA | qRT-PCR |
|  | R: GTCACACGCATGTTCCCAAG | qRT-PCR |
| CCL4 primers | F: GCTTCCTCGCAACTTTGTGG | qRT-PCR |
|  | R: TCACTGGGATCAGCACAGAC | qRT-PCR |
| CCL5 primers | F: GACAGCAAGTCTGGCAGGAT | qRT-PCR |
|  | R: TTTTGACAAAGCAGCGCCTC | qRT-PCR |
| CCL19 primers | F: GAAGACTGCTGCCTGTCTGT | qRT-PCR |
|  | R: GCAGTCTCTGGATGATGCG | qRT-PCR |
| CCL21 primers | F: AAGGAAGATTCCCGCCAAGG | qRT-PCR |
|  | R: TGTACTGGGGAGCCGTATCA | qRT-PCR |
| CXCL9 primers | F: TTAGCATGCTGGTGAGCCAA | qRT-PCR |
|  | R: GCCCTCAAGGAGCTGACAAT | qRT-PCR |
| CXCL10 primers | F: GCCATTCTGATTTGCTGCCT | qRT-PCR |
|  | R: GCAGGTACAGCGTACAGTTCT | qRT-PCR |
| CXCL11 primers | F: TGTCTTTGCATAGGCCCTGG | qRT-PCR |
|  | R: GACTCCTTTGGGCAGTGGAA | qRT-PCR |
| circIGF2BP3 plasmid primers | F: CGGAATTCTGAAATATGCTATCTTACAGGTGCTGGATAGTTTACTAGTCCAGT | plasmid construction |
|  | R: CGGGATCCTCAAGAAAAAATATATTCACCGTTTTGCCTCCTTTTCCAATAACT | plasmid construction |
| circIGF2BP3-si-Mut plasmid primers | F: CGGAATTCTGAAATATGCTATCTTACAGGGATGGCTATGTTTACTAGTCCAGT | plasmid construction |
|  | R: CGGGATCCTCAAGAAAAAATATATTCACCGCGAAGCGTCCTTTTCCAATAACT | plasmid construction |
| OTUB1 promoter fragment primers | F: GGCCTCTCCTACAGGTCTCA | luciferase |
|  | R: GGATCCCCTTAGGCGTTGTC | luciferase |
| IGF2BP3 promoter fragment primers | F: GTTATCTGGTCTGGGCGGAT | luciferase |
|  | R: CCCCGACAACCACCATTGTA | luciferase |

**Supplementary Table S8.** Correlation between circIGF2BP3 levels and different clinical characteristics of NSCLC in cohort I.

| **Characteristics** | **circIGF2BP3 Expression** | | **P value** |
| --- | --- | --- | --- |
|  | **Low** | **High** |  |
| Sex |  |  |  |
| Male | 14 | 27 | 0.4156 |
| Female | 6 | 21 |  |
| Age |  |  |  |
| ≤60 | 13 | 29 | 0.7896 |
| >60 | 7 | 19 |  |
| Tumor invasion depth |  |  |  |
| T1-T2 | 16 | 18 | **0.0029** |
| T3-T4 | 4 | 30 |  |
| Lymph node metastasis |  |  |  |
| N0 | 13 | 17 | **0.0334** |
| N1-N3 | 7 | 31 |  |
| TNM Stage |  |  |  |
| I- II | 15 | 21 | **0.0317** |
| III-IV | 5 | 27 |  |

Statistical analysis was performed using Fisher’s exact test. P values <0.05 were considered significant.
